# Meditation, free-will beliefs, and sentencing attitudes: an exploratory pilot study in India

**DOI:** 10.3389/fpsyg.2026.1865426

**Published:** 2026-08-10

**Authors:** Pragya Mishra, Susan L. Prescott, Alan C. Logan

**Affiliations:** 1 Department of Law, University of Allahabad, Prayagraj, Uttar Pradesh, India; 2 Center for Justice and Mental Well-Being, Nova Institute for Health, Baltimore, MD, United States; 3 School of Medicine, University of Western Australia, Perth, WA, Australia; 4 Department of Family and Community Medicine, University of Maryland School of Medicine, Baltimore, MD, United States

**Keywords:** Anapana, criminal justice, dehumanization, free-will beliefs, meditation, moral foundations, rehabilitation, sentencing attitudes

## Abstract

Criminal sentencing attitudes can reflect psychological dispositions concerning agency, hierarchy, blame, and rehabilitation. This exploratory pilot examined whether established contemplative practice is associated with a less punitive profile and whether legal professionals show differences following a four-week Anapana programme. Ninety adults participated in three cohorts: experienced meditators (*n* = 30), meditation-naïve controls (*n* = 30), and legal professionals or advanced law trainees assessed before and after the programme (*n* = 30). The study assessed 21 outcomes: 15 core outcomes covering anxiety, free-will beliefs, aggression, social dominance, just-world beliefs, pretrial juror attitudes, and vignette-based sentencing judgments, plus six additional exploratory outcomes comprising dehumanization and five moral-foundation subscales. The six exploratory outcomes, initially envisaged for a subsequent study, were incorporated during rolling data collection; Group C completed them before beginning the programme and again at post-assessment. All 21 omnibus group comparisons remained significant after false-discovery-rate and Bonferroni correction. The experienced-meditator versus control differences also remained significant after adjustment for exact age and in a sensitivity analysis restricted to the shared 50–60-year age range. Following the programme, Group C showed statistically significant pre–post differences on all 21 outcomes (|*d*_*z*| = 1.05–3.11). The direction, coherence, and timing of those differences are consistent with a meaningful meditation-related contribution, although the uncontrolled design cannot estimate how much was attributable specifically to Anapana rather than expectancy, repeated testing, demand characteristics, regression to the mean, or other time-related influences. Exploratory indirect-effect analyses did not establish causal mechanisms: only the anxiety-to-aggression model was directionally consistent, while an aggression-to-rehabilitation model showed inconsistent mediation or suppression. No retreat contrast survived multiplicity correction. The findings are therefore best interpreted as hypothesis-generating evidence of a coherent pattern that warrants preregistered, adequately powered randomized trials with concurrently reassessed active controls, behavioural outcomes, and follow-up assessment.

## 1 Introduction

Criminal sentencing lies at the intersection of law, morality, and psychology. Although formal doctrine directs attention to culpability, harm, proportionality, and mitigation, sentencing judgments are also made within cultural and psychological environments that shape beliefs about agency, blame, hierarchy, and rehabilitation. Cross-national incarceration rates differ markedly, but those differences reflect many institutional, economic, political, and historical influences; they should not be attributed to any single cultural characteristic. India nevertheless offers a valuable non-WEIRD context in which to examine how contemplative traditions and punitive attitudes coexist.

Research with judges, mock jurors, and community samples shows that legally relevant judgments can vary with dispositional aggression, social dominance orientation, beliefs about the fixedness of criminal behaviour, and racialized or other group-based biases (Guthrie et al., 2007; Rachlinski et al., 2009; Berryessa, 2018). Vignette methods cannot reproduce real sentencing, but they permit controlled examination of responsibility and punishment judgments and are widely used in legal psychology (Sporer and Goodman-Delahunty, 2009; Berryessa, 2018).

## 2 Free-will beliefs and punitive attitudes

Beliefs about human agency are relevant to punishment because stronger endorsement of unconstrained choice is often associated with blame and retributive preferences (Carey and Paulhus, 2013; Genschow et al., 2017; Genschow and Vehlow, 2021; Walton et al., 2026). Experimental studies also suggest that mechanistic accounts of behaviour and motivated reasoning can influence free-will belief and punishment judgments (Shariff et al., 2014; Clark et al., 2014). Recent cross-national evidence from Norway, Sweden, and the United Kingdom further indicates that national context covaries with both free-will beliefs and punishment severity, and that stronger free-will endorsement is associated with harsher punishment across the combined sample (Solum et al., 2026). Some scholars argue that legal practice relies too heavily on commonsense assumptions about choice and control, while compatibilist accounts maintain that responsibility can remain meaningful without metaphysical indeterminism (Atiq, 2013; Morse, 2007). The present study does not attempt to settle that philosophical debate. It asks the narrower empirical question whether free-will and determinism beliefs covary with meditation experience and vignette-based sentencing attitudes.

## 3 Social dominance, just-world beliefs, and dehumanization

Social dominance orientation (SDO) reflects preference for intergroup hierarchy and has been associated with discriminatory attitudes, reduced concern for equality, and support for harsh sanctions (Pratto et al., 1994; Sidanius et al., 2006; Ho et al., 2015). Belief in a just world (BJW) can support the view that people receive outcomes they deserve and has been linked to victim derogation and punitive judgments (Jost et al., 2004; Hafer and Sutton, 2016). Dehumanization, or diminished attribution of full humanity to others, is likewise associated with punishment and sentence severity (Kteily et al., 2015; Kteily and Landry, 2022; Landry et al., 2024). These constructs provide complementary, but substantially intercorrelated, indicators of punitive orientation.

## 4 Contemplative practice and moral judgment

Mindfulness and related contemplative practices have been associated with reduced anxiety and aggression, increased empathic concern, and changes in prejudice or social-dominance measures (Khoury et al., 2013; Goldberg et al., 2018; Parks et al., 2014; Panno et al., 2018; Gillions et al., 2019). Anapana, or observation of natural respiration, is commonly taught as a preparatory attentional practice within the Goenka Vipassana tradition. It trains sustained attention and nonjudgmental return to the breath when distraction occurs. Preliminary research has also linked intensive meditation experience with lower free-will endorsement (Sabugo et al., 2024). The conceptual relationship between conditioned agency, contemplative experience, and criminal responsibility has also been developed in prior Indian legal and interdisciplinary scholarship (Mishra, 2018; Logan et al., 2026).

These findings do not imply that contemplative practice necessarily produces more just legal decisions. At the same time, rehabilitation-oriented sentencing should not be equated with an absence of accountability or with unstructured leniency: it can include proportionate sanctions, public-safety conditions, victim-sensitive processes, treatment, supervision, and reintegration. Conversely, lower punitive severity cannot by itself establish a better justice outcome. The present study therefore treats rehabilitation as a legitimate sentencing orientation and an outcome of empirical interest, not as proof of normative superiority. Individual-level training may also be co-opted as a substitute for institutional reform, a concern captured by the “McMindfulness” critique (Purser, 2019). Any potential role for contemplative education in legal settings must therefore be evaluated as a complement to, not a replacement for, structural reforms addressing sentencing law, inequality, prosecutorial power, carceral conditions, and access to mitigation evidence.

## 5 The current study

Three gaps motivated this pilot. First, little research has combined a cross-sectional comparison of experienced meditators with an intervention arm focused on sentencing attitudes. Second, most evidence on free-will beliefs, dehumanization, moral foundations, and punishment comes from Western samples. Third, candidate pathways are often studied separately rather than within a common battery. We therefore compared experienced meditators, meditation-naïve controls, and legal professionals at baseline, and we assessed the legal-professional cohort again after a four-week Anapana programme. The cross-sectional arm was associational; the intervention arm was an uncontrolled within-subject pre–post evaluation. Both were intended to generate hypotheses for a later randomized study, not to establish causality.

## 6 Methods

### 6.1 Participants and recruitment

Ninety adults participated across three cohorts. Group A comprised experienced Vipassana or Anapana meditators (*n* = 30) recruited through meditation networks; participants reported approximately 10–20 or more years of sustained practice and multiple extended retreats. Group B comprised meditation-naïve adults (*n* = 30) recruited through local community and professional networks. Group C comprised legal professionals and advanced law trainees, including some serving judicial magistrates in the subordinate judiciary (*n* = 30), recruited through legal-professional networks for the four-week programme. Recruitment was rolling in Prayagraj and surrounding areas, and no participants were recruited from the principal investigator’s own classes or direct supervisees. Invitations were circulated through professional, community, and contemplative networks and could be forwarded. Because the total number of unique individuals who received or viewed an invitation was not recorded, a recruitment response rate could not be calculated.

Participation was voluntary and was not contingent on payment. Light refreshments and modest non-monetary hospitality were provided during in-person sessions. Most participants declined monetary compensation; experienced meditators generally did so for service-oriented reasons, while legal professionals generally did so in keeping with professional requirements. No compensation was linked to questionnaire responses, intervention adherence, study completion, or study outcomes.

Exact ages were available for all participants. Group A had a mean age of 55.13 years (SD = 3.01; median = 55; range = 50–60), Group B 51.10 years (SD = 8.17; median = 53.5; range = 31–60), and Group C 33.00 years (SD = 3.21; median = 32; range = 30–44). Twenty-two of the 30 Group B participants were aged 50–60, providing substantial age overlap with Group A. Gender distribution was approximately balanced, all participants had university-level education, and materials were available in English and Hindi. Post-intervention assessments used the same language selected at baseline.

Ethics approval was granted by the PSCRC Institutional Ethics Committee, Agra, on 20 November 2025 under Indian Council of Medical Research guidelines. All participants provided written informed consent. There was zero attrition, and no participant-level scale scores were missing in the analysis files (Table 1).

**Table 1:** Cohort characteristics and complete descriptive statistics.

Characteristic/outcome	Group A	Group B	Group C pre	Group C post
*n*	30	30	30	30
Age, *M* (SD)	55.13 (3.01)	51.10 (8.17)	33.00 (3.21)	Same cohort
Age, median [range]	55 [50–60]	53.5 [31–60]	32 [30–44]	Same cohort
STAI-6 (anxiety)	8.40 (1.52)	16.80 (2.20)	15.80 (1.61)	12.60 (1.10)
FWI free will	22.30 (4.11)	28.70 (2.56)	27.50 (1.94)	25.30 (0.95)
FWI determinism	24.50 (3.25)	15.50 (1.83)	16.80 (1.27)	18.70 (1.44)
FWI dualism	26.50 (2.36)	23.30 (2.32)	22.30 (1.73)	23.50 (2.11)
BPAQ-SF (aggression)	23.30 (5.10)	35.70 (2.71)	31.20 (2.31)	27.10 (1.77)
SDO-7	37.60 (7.27)	62.50 (4.00)	59.50 (3.61)	54.20 (2.71)
BJW	22.50 (2.60)	33.00 (2.59)	29.30 (2.51)	27.90 (1.88)
PJAQ system confidence	15.30 (1.70)	24.00 (1.93)	26.50 (2.03)	24.60 (1.28)
PJAQ cynicism	15.20 (1.63)	22.70 (2.07)	20.30 (2.09)	18.40 (1.33)
PJAQ racial bias	8.60 (1.79)	15.30 (1.86)	14.60 (1.57)	12.80 (1.32)
PJAQ social justice	15.70 (1.68)	10.80 (1.61)	12.50 (1.43)	13.70 (1.66)
PJAQ innate criminality	7.60 (1.69)	17.50 (1.76)	14.80 (1.94)	12.40 (1.33)
RRI	4.40 (0.61)	2.80 (0.39)	3.20 (0.29)	4.10 (0.58)
Incarceration	1.70 (0.77)	4.70 (0.65)	3.60 (0.57)	2.80 (0.39)
Responsibility	4.20 (0.58)	5.60 (0.33)	5.40 (0.35)	4.90 (0.21)
Dehumanization	13.97 (5.19)	33.77 (6.32)	32.43 (7.99)	20.57 (8.48)
MFQ care	25.20 (2.28)	19.63 (2.47)	19.23 (2.10)	22.63 (2.58)
MFQ fairness	24.63 (2.30)	19.53 (2.36)	19.90 (2.06)	22.57 (1.83)
MFQ loyalty	12.00 (1.89)	16.97 (2.61)	17.23 (2.39)	13.73 (2.73)
MFQ authority	12.83 (1.91)	17.20 (2.02)	16.93 (2.53)	13.77 (2.69)
MFQ purity	11.50 (1.81)	16.77 (2.69)	16.57 (2.84)	12.87 (3.14)

Outcome entries are *M* (SD). Group C post comprises the same 30 participants reassessed after the four-week programme. Gender distribution was approximately balanced across cohorts, and all participants had university-level education. Detailed inferential comparisons are reported in Table 3 and Supplementary Table S1.

### 6.2 Design and assessment sequence

The mixed design combined cross-sectional baseline comparisons among Groups A, B, and C-pre with an uncontrolled within-subject pre–post evaluation in Group C. Six Group C participants independently elected to attend a 10-day Vipassana retreat during the four-week period; they were identified from attendance records and analysed only as a small, self-selected exploratory subgroup.

**Figure 1:**
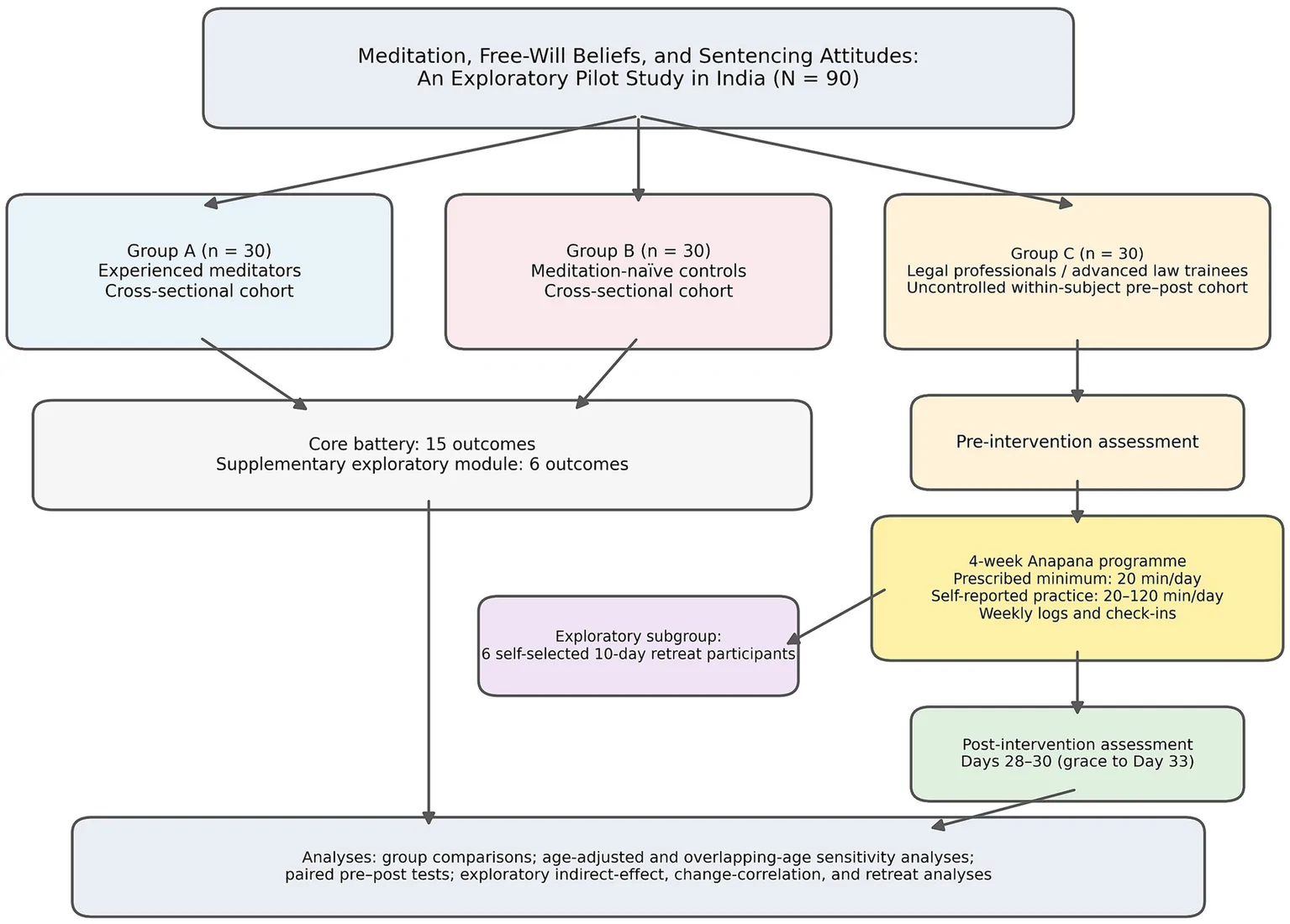
Study design and sample flow. Group B served as a cross-sectional comparison cohort, not as a concurrent control for Group C. The prescribed intervention minimum was 20 min daily; longer practice was participant-initiated.

The fixed core-battery sequence was: demographic profile; STAI-6; Free Will Inventory; BPAQ-SF; SDO-7; BJW; PJAQ; and four sentencing vignettes. The later exploratory module comprised the Ascent dehumanization rating and MFQ-30. Standardizing the sequence reduced procedural variation, but order and repeated-measure effects were not independently estimated (Figure 1).

### 6.3 Core battery

State anxiety was measured with the STAI-6. All six 1–4 item responses were scored in the anxiety direction and summed without prorating or conversion to the conventional 20–80 STAI metric, yielding a raw total from 6 to 24; higher scores indicate greater state anxiety (Marteau and Bekker, 1992). Agency beliefs were assessed with the Free Will Inventory subscales for Free Will, Determinism, and Dualism (Nadelhoffer et al., 2014). Aggression was measured using the BPAQ-SF (Bryant and Smith, 2001); social dominance with SDO-7 (Ho et al., 2015); and just-world beliefs with the 7-item BJW scale (Lipkus, 1991). The PJAQ yielded System Confidence, Cynicism, Racial Bias, Social Justice, and Innate Criminality subscales (Lecci and Myers, 2008). Validated scale structures were used; no exploratory factor analysis was conducted.

Sentencing judgments were assessed through four hypothetical criminal-case vignettes. For each vignette, participants recommended incarceration duration (0–10 years), attributed responsibility (1–7), and selected a disposition ranging from custodial to rehabilitative or restorative options. Scores were averaged across cases to form incarceration duration, responsibility attribution, and a Rehabilitation/Restorative Index (RRI). The full vignettes and participant-facing instructions are reproduced in the Supplementary material. These are vignette-based attitudes, not observed judicial behaviour.

### 6.4 Exploratory supplementary measures

The Ascent of Man dehumanization scale (0–100) and the five MFQ-30 subscales—Care, Fairness, Loyalty, Authority, and Purity—were incorporated as an exploratory supplementary module (Kteily et al., 2015; Graham et al., 2011). The module was initially envisaged for a subsequent study, but was added to the ongoing pilot while rolling data collection was still in progress. For Group C, each participant completed the supplementary module before commencing the four-week programme and completed it again at post-intervention assessment. Groups A and B completed the module once. These six outcomes were not treated as preregistered confirmatory endpoints.

### 6.5 Four-week Anapana programme

Participants attended an in-person orientation delivered by a specialised meditation teacher. They received simplified written instructions based on the Goenka-tradition Anapana method and audio guidance. Initial guided recordings lasted approximately 20–25 min, and later reinforcement recordings lasted approximately 10 min. The core instruction was to observe the natural breath without deliberately changing it, notice distraction without self-criticism, and gently return attention to respiration. The programme prescribed at least 20 min of daily practice for 4 weeks; participants were free to practice longer (Table 2).

**Table 2:** Structure and delivery of the four-week Anapana Programme.

Component	Implemented procedure
Orientation	One in-person session delivered by a specialised meditation teacher before home practice began.
Instructional content	Observation of natural respiration; nonjudgmental recognition of distraction; gentle return of attention to the breath.
Materials	Written instructions and Goenka-tradition Anapana audio guidance; initial recordings approximately 20–25 min, reinforcement recordings approximately 10 min.
Prescribed dose	Minimum 20 min daily for 4 weeks; longer practice was permitted but not required.
Monitoring	Self-report practice logs collected weekly and weekly check-ins used to reinforce technique, answer practical questions, and support adherence.
Completion criterion	Attendance at orientation; at least 20 min of daily practice; submission of all four weekly logs; participation in all weekly check-ins; and post-assessment by Day 33.
Completion	All 30 participants fulfilled every completion criterion.
Observed dose	Self-reported practice ranged from 20 to approximately 120 min daily; 24 participants reported practice at or near 60 min twice daily by the final week.
Retreat subgroup	Six participants self-selected into a 10-day Vipassana retreat during the four-week period; retreat status was not assigned or randomized.

### 6.6 Statistical analysis

One-way ANOVAs compared Groups A, B, and C-pre. Effect sizes are reported as eta-squared and pairwise Cohen’s d. Paired t tests evaluated Group C pre–post changes, with mean-change 95% confidence intervals and within-subject Cohen’s *d*_*z*. Because 21 outcomes were examined, Benjamini–Hochberg false-discovery-rate q values were calculated across the 21 omnibus tests and across the 21 paired tests. A conservative Bonferroni threshold of 0.05/21 = 0.00238 was also used as a sensitivity benchmark.

Age robustness was examined in two ways. First, Group A versus Group B contrasts were estimated with exact age as a covariate using HC3-robust Wald tests and 95% confidence intervals. Age-by-group interactions were tested for all 21 outcomes. Second, a conservative sensitivity analysis compared all Group A participants with the 22 Group B participants in the shared 50–60-year age range, providing an assumption-light complement where common age slopes were questionable. Age-adjusted A–C contrasts were treated cautiously because those cohorts had little common age support. Within Group B, both Pearson and Spearman age–outcome correlations were examined; within Group C, age was correlated with pre–post change. Correlation families were FDR-adjusted across all 21 outcomes.

Gap closure was calculated descriptively as (|C-pre − A| − |C-post − A|)/|C-pre − A| × 100. It benchmarks movement toward the observed Group A mean and is not an estimate of the proportion of a causal meditation effect achieved.

Eighteen single-mediator models were re-estimated in the Group A–B sample using standardized coefficients and 20,000 bias-corrected bootstrap resamples with a fixed random seed (1865426) for reproducibility. Total, direct, and indirect effects are reported. Because these models are cross-sectional, they are presented only as exploratory indirect-effect descriptions. Retreat analyses regressed standardized post-scores on retreat status and baseline score with HC3-robust confidence intervals; all 21 retreat *p* values were FDR-adjusted. The full sample remained the primary analysis; C21 was examined only in a *post hoc* leave-one-participant-out sensitivity analysis because this participant showed limited change across the core battery.

## 7 Results

### 7.1 Age distribution and robustness

**Figure 2:**
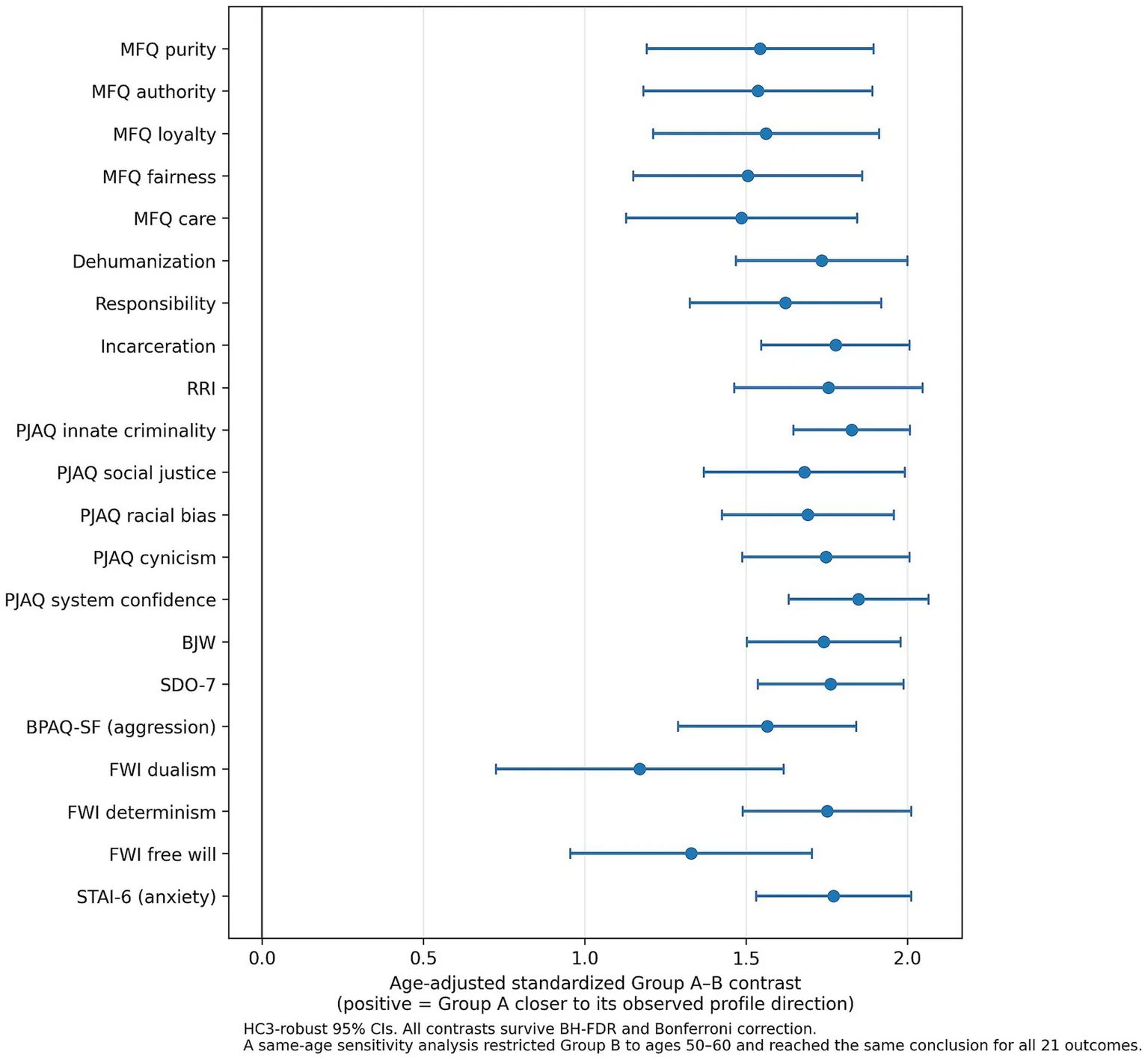
Age-adjusted Group A–B contrasts across 21 outcomes. Outcomes were sign-aligned so that positive contrasts indicate a difference in the direction observed in Group A (for example, lower anxiety and higher determinism endorsement). Error bars are HC3-robust 95% confidence intervals. All contrasts survived FDR and Bonferroni correction; same-age sensitivity analyses reached the same conclusion. Common-slope estimates for aggression and responsibility attribution require caution because their age-by-group interactions survived FDR correction.

Age differed across the three cohorts, *F*(2, 87) = 145.30, *p* < 0.001, principally because Group C was younger. The Group A–B mean difference was smaller and the distributions overlapped substantially: 22 of 30 controls were aged 50–60, the complete age range of Group A. Every Group A–B difference across the 21 outcomes remained significant after exact-age adjustment with HC3-robust inference and after FDR and Bonferroni correction. Every difference also remained significant in the overlapping-age sensitivity analysis restricted to ages 50–60 (same-age |*d*| range = 1.37–5.60; all *p* < 0.00238). Age-by-group interactions survived FDR correction for aggression (*q* = 0.015) and responsibility attribution (*q* = 0.047), so the common-slope adjusted estimates for those outcomes are interpreted cautiously; both remained strongly different in the same-age restricted analysis. Within Group B, no age–outcome association survived FDR correction. Within Group C, no age–change association survived correction (Supplementary Tables S2, S5A–S5B). These results reduce, but do not eliminate, concern that the Group A–B pattern is solely an age artefact (Figure 2).

### 7.2 Between-group comparisons

All 21 omnibus comparisons among Groups A, B, and C-pre were significant after FDR correction and below the Bonferroni threshold (eta-squared range = 0.417–0.869). Group A differed from Group B across affective, agency-belief, dispositional, legal-attitude, sentencing, dehumanization, and moral-foundation outcomes. The size and uniformity of these differences are unusual and should be interpreted alongside self-selection, common-method variance, restricted distributions, and other unmeasured group characteristics. Complete means, standard deviations, exact *p* values, effect sizes, and age-robustness analyses are reported in Supplementary Tables S1–S2; full distributions appear in Supplementary Figures S1–S2. The descriptive full-profile heatmap is retained as Supplementary Figure S6.

### 7.3 Pre–post changes in group C

**Figure 3:**
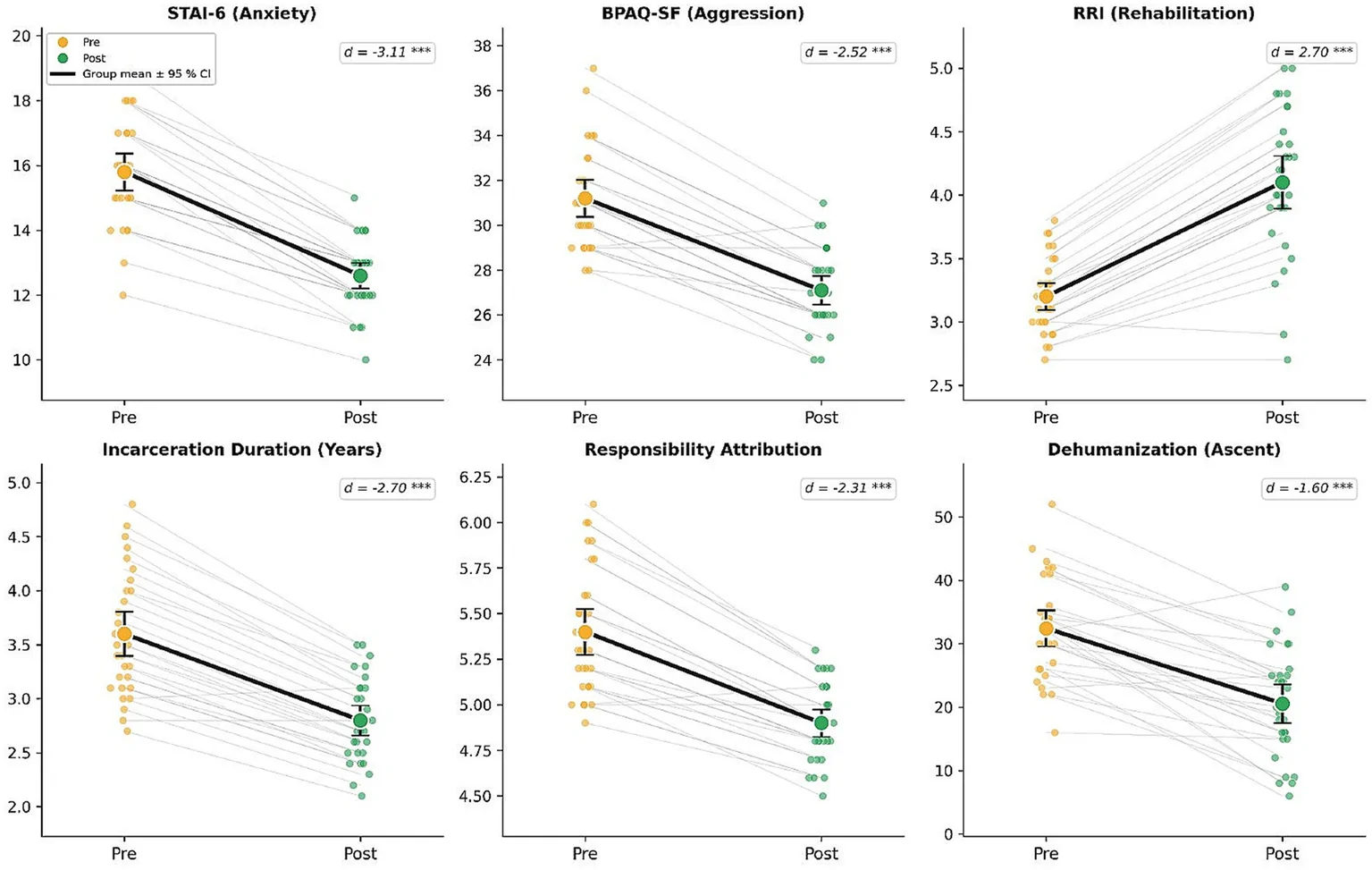
Individual Group C pre–post scores for six representative outcomes. Grey lines connect each participant’s scores; large points and error bars show group means and 95% confidence intervals. *d_z* is paired Cohen’s *d*. The figure displays observed change and does not establish programme-specific causation.

Group C showed statistically significant pre–post changes on all 21 outcomes after FDR correction and below the Bonferroni threshold. Absolute *d*_*z* values ranged from 1.05 to 3.11. These are large effects relative to the moderate average effects commonly reported in mindfulness meta-analyses, and the discrepancy warrants caution rather than stronger causal attribution. The same participants completed all measures, so age cannot account for change over 4 weeks. The direction, consistency across outcomes, and temporal alignment with the programme are compatible with a meaningful meditation-related contribution; however, expectancy, social desirability, repeated testing, history, regression to the mean, and demand characteristics remain viable alternative explanations (Figure 3, Table 3).

**Table 3:** Group C pre–post changes across 21 outcomes.

Outcome	Pre *M* (SD)	Post *M* (SD)	Mean Δ	95% CI for Δ	*t*(29)	FDR q	*d*_*z*	Gap %
STAI-6 (anxiety)	15.80 (1.61)	12.60 (1.10)	−3.20	[−3.58, −2.82]	−17.01	<0.001	−3.11	43.2
FWI free will	27.50 (1.94)	25.30 (0.95)	−2.20	[−2.64, −1.76]	−10.16	<0.001	−1.85	42.3
FWI determinism	16.80 (1.27)	18.70 (1.44)	+1.90	[+1.57, +2.23]	11.76	<0.001	+2.15	24.7
FWI dualism	22.30 (1.73)	23.50 (2.11)	+1.20	[+0.92, +1.48]	8.64	<0.001	+1.58	28.6
BPAQ-SF (aggression)	31.20 (2.31)	27.10 (1.77)	−4.10	[−4.71, −3.49]	−13.81	<0.001	−2.52	51.9
SDO-7	59.50 (3.61)	54.20 (2.71)	−5.30	[−6.07, −4.53]	−14.14	<0.001	−2.58	24.2
BJW	29.30 (2.51)	27.90 (1.88)	−1.40	[−1.79, −1.01]	−7.39	<0.001	−1.35	20.6
PJAQ system confidence	26.50 (2.03)	24.60 (1.28)	−1.90	[−2.24, −1.56]	−11.28	<0.001	−2.06	17.0
PJAQ cynicism	20.30 (2.09)	18.40 (1.33)	−1.90	[−2.32, −1.48]	−9.25	<0.001	−1.69	37.3
PJAQ racial bias	14.60 (1.57)	12.80 (1.32)	−1.80	[−2.13, −1.47]	−11.12	<0.001	−2.03	30.0
PJAQ social justice	12.50 (1.43)	13.70 (1.66)	+1.20	[+0.95, +1.45]	9.89	<0.001	+1.81	37.5
PJAQ innate criminality	14.80 (1.94)	12.40 (1.33)	−2.40	[−2.79, −2.01]	−12.67	<0.001	−2.31	33.3
RRI	3.20 (0.29)	4.10 (0.58)	+0.90	[+0.78, +1.02]	14.79	<0.001	+2.70	75.0
Incarceration	3.60 (0.57)	2.80 (0.39)	−0.80	[−0.91, −0.69]	−14.81	<0.001	−2.70	42.1
Responsibility	5.40 (0.35)	4.90 (0.21)	−0.50	[−0.58, −0.42]	−12.65	<0.001	−2.31	41.7
Dehumanization	32.43 (7.99)	20.57 (8.48)	−11.87	[−14.64, −9.10]	−8.76	<0.001	−1.60	64.3
MFQ care	19.23 (2.10)	22.63 (2.58)	+3.40	[+2.31, +4.49]	6.35	<0.001	+1.16	57.0
MFQ fairness	19.90 (2.06)	22.57 (1.83)	+2.67	[+1.73, +3.60]	5.82	<0.001	+1.06	56.3
MFQ loyalty	17.23 (2.39)	13.73 (2.73)	−3.50	[−4.61, −2.39]	−6.43	<0.001	−1.17	66.9
MFQ authority	16.93 (2.53)	13.77 (2.69)	−3.17	[−4.29, −2.04]	−5.77	<0.001	−1.05	77.2
MFQ purity	16.57 (2.84)	12.87 (3.14)	−3.70	[−5.02, −2.38]	−5.75	<0.001	−1.05	73.0

Gap % is descriptive movement toward the observed Group A mean; it is not a causal efficacy estimate. All raw *p* values and FDR q values were below 0.001 and below the Bonferroni threshold of 0.00238; exact values are retained in the analysis files.

### 7.4 Descriptive gap closure and sentencing triad

**Figure 4:**
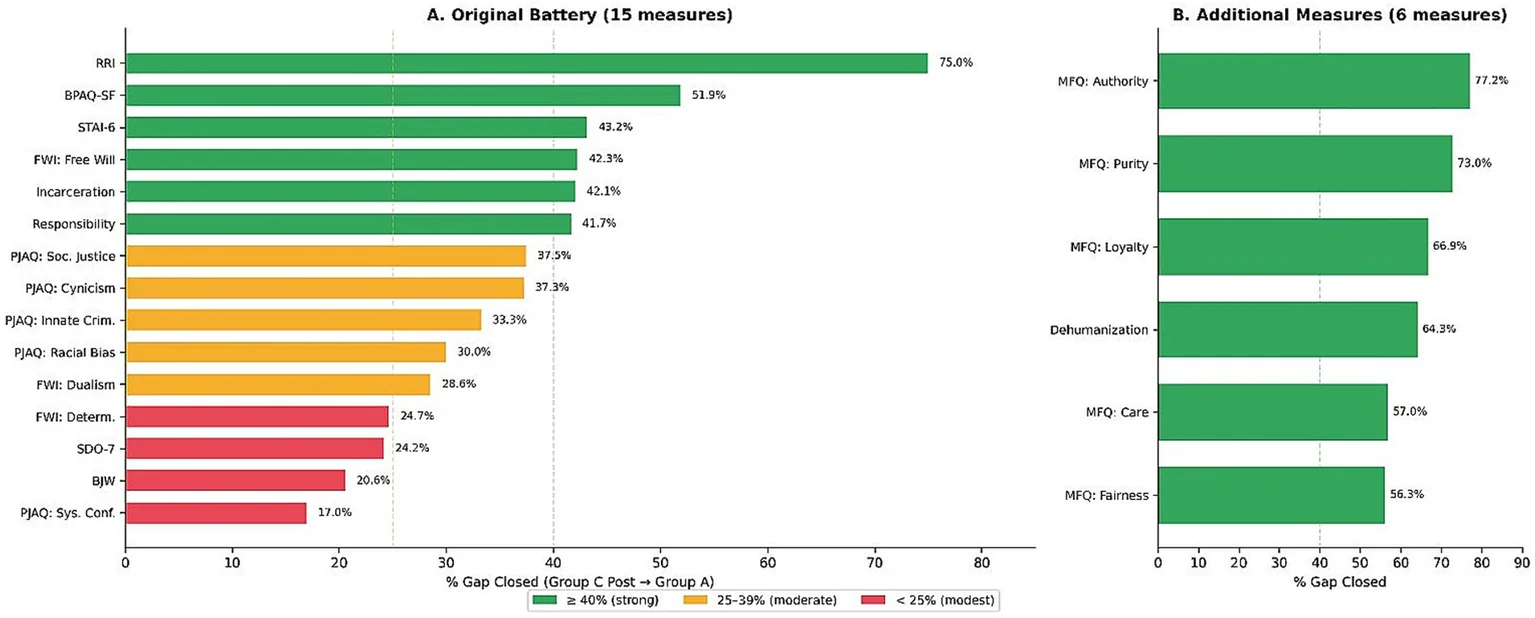
Descriptive gap closure toward the observed Group A mean. The metric indicates profile convergence and should not be interpreted as the proportion of a causal intervention effect achieved.

**Figure 5:**
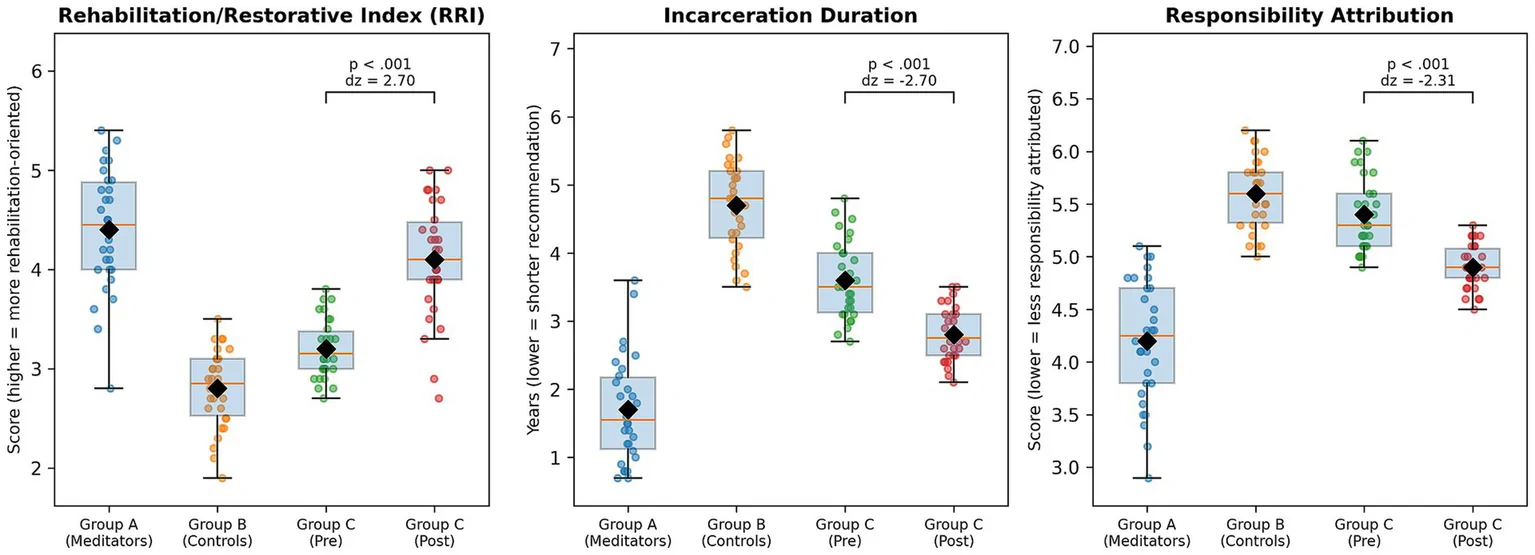
Sentencing-triad group comparisons. Box plots show distributions, points show individual observations, and diamonds show means. Brackets summarize paired Group C pre–post tests. Outcomes are vignette-based judgments rather than real sentencing behaviour.

Gap-closure values ranged from 17.0 to 77.2%. The largest values were observed for MFQ Authority, RRI, MFQ Purity, MFQ Loyalty, dehumanization, MFQ Care, and MFQ Fairness. Because Group A is a self-selected cross-sectional cohort with decades of practice, these values are best understood as descriptive profile convergence, not as a dose–response curve or a percentage of treatment efficacy (Figures 4, 5).

### 7.5 Exploratory indirect effects and change associations

Two of 18 fixed-seed, bias-corrected bootstrap indirect-effect intervals excluded zero. In the STAI-6 → BPAQ-SF model, the indirect effect was directionally consistent with the total group difference (indirect = −0.371, 95% CI [−0.645, −0.049]), but the outcome was aggression rather than sentencing. In the BPAQ-SF → RRI model, the indirect effect was opposite in sign to the total effect (indirect = −0.368, 95% CI [−0.610, −0.133]; total = +0.845; direct = +1.213), indicating inconsistent mediation or suppression rather than a straightforward mechanism. For SDO-7 → RRI, the upper confidence bound was approximately zero (indirect = −0.249, 95% CI [−0.551, +0.004]), so the effect could not be reliably distinguished from zero. No serial chain was interpreted as evidence of parallel or sequential causation. Full decompositions appear in Supplementary Table S3.

**Figure 6:**
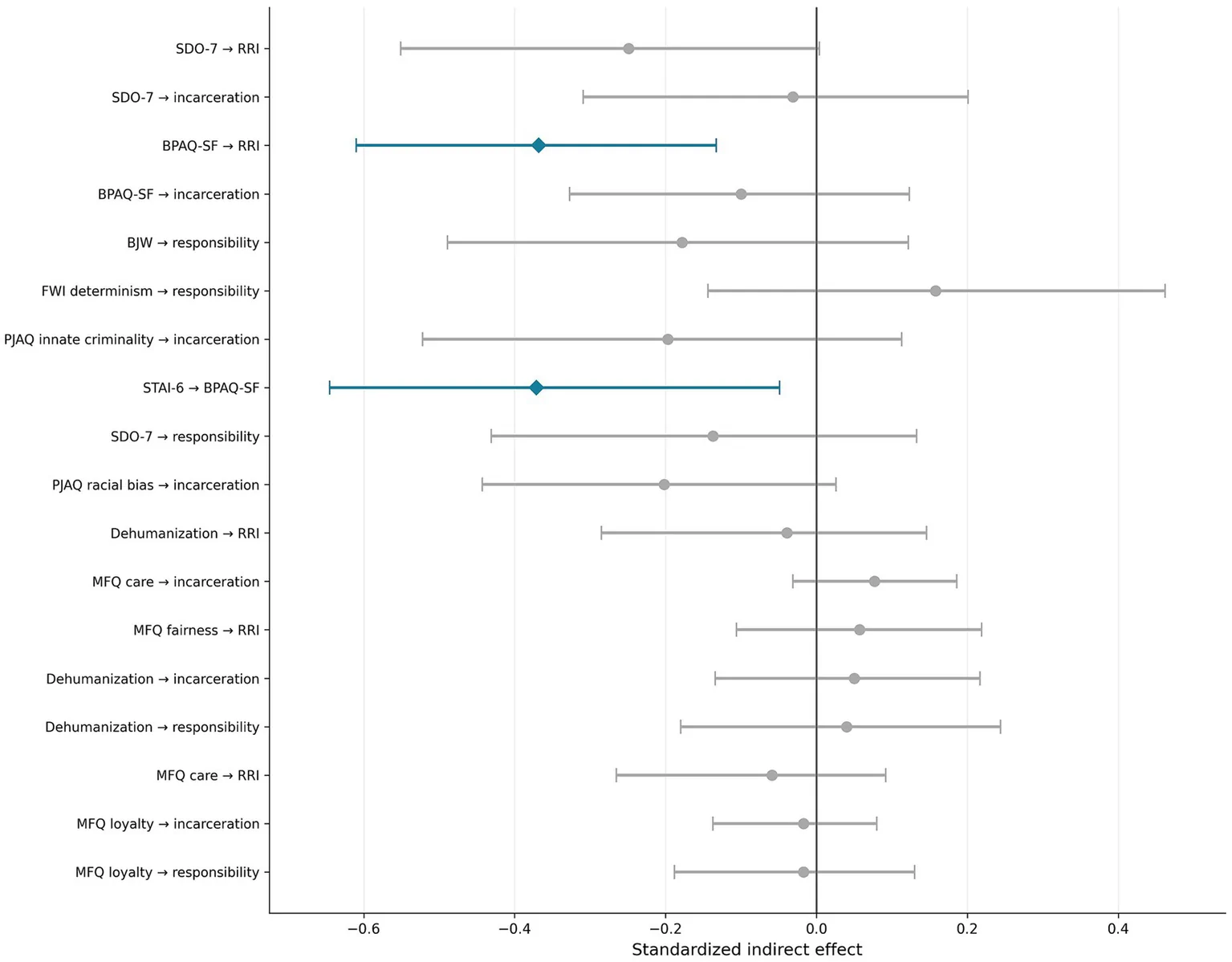
Exploratory Group A–B indirect-effect models. Points are standardized indirect effects and lines are fixed-seed, bias-corrected bootstrap 95% confidence intervals based on 20,000 resamples. Models are cross-sectional and hypothesis-generating; highlighted results must not be interpreted causally.

Within Group C, changes in several outcomes were strongly correlated, particularly aggression, innate-criminality beliefs, RRI, incarceration, and responsibility. These associations describe coordinated change but do not establish temporal mediation because all change scores cover the same interval. The complete change-correlation matrix appears in Supplementary Figure S4 (Figure 6).

### 7.6 Participant sensitivity and retreat subgroup

The full *N* = 30 Group C sample was retained for all primary pre–post analyses. A *post hoc* sensitivity analysis excluding C21, who showed little or no change on most core outcomes, generally strengthened rather than created the effect estimates; all conclusions were unchanged. Supplementary Figure S5 reports the percentage of outcomes moving in the hypothesised direction for each participant without labelling below-average participants as treatment non-responders.

**Figure 7:**
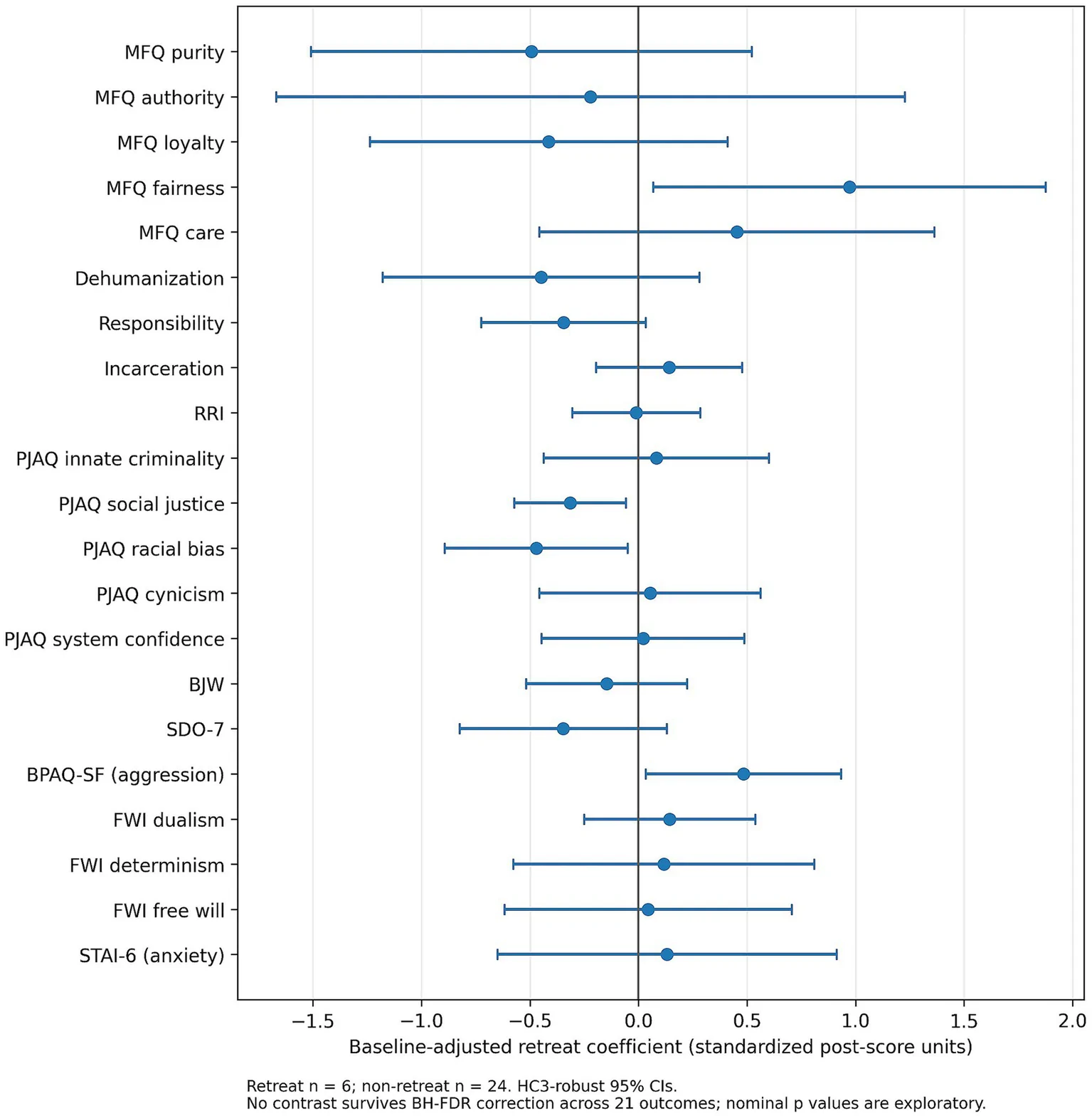
Baseline-adjusted retreat versus non-retreat contrasts across 21 outcomes. Error bars are HC3-robust 95% confidence intervals. No contrast survived FDR correction; all results are exploratory.

Six participants self-selected into an additional 10-day retreat. Several baseline-adjusted retreat coefficients had nominal *p* values below 0.05, including MFQ Fairness, but no contrast survived FDR correction across 21 outcomes (minimum *q* = 0.186). The subgroup is too small and non-randomized for efficacy conclusions. Complete baseline-adjusted estimates appear in Supplementary Table S4, and participant-level slopes appear in Supplementary Figures S3A–S3B (Figure 7).

## 8 Discussion

### 8.1 Principal findings

Two converging descriptive patterns were observed. Experienced meditators differed from meditation-naïve controls across the 21-outcome battery, and every A–B contrast remained statistically significant after exact-age adjustment and in the shared-age sensitivity analysis. Group C also displayed coordinated pre–post differences after the four-week Anapana programme. Their direction, coherence, and temporal alignment are consistent with a meaningful meditation-related contribution, but the design cannot quantify the proportion attributable specifically to Anapana rather than repeated testing, expectancy, time, or other influences.

The effect sizes were substantially larger than the moderate averages commonly reported in mindfulness meta-analyses (Khoury et al., 2013; Goldberg et al., 2018). A randomized Anapanasati study reported lower verbal aggression, but it does not provide a benchmark for the unusually broad pattern observed here (Sivaramappa et al., 2019). Plausible contributors include self-selection, intensive self-reported practice, restricted variance, common-method bias, social desirability, expectancy, and correlated outcomes. Distributional checks and multiplicity correction reduce some concerns but do not substitute for randomization, blinding, and an interval-matched active control.

### 8.2 Age, intervention interpretation, and ecological validity

Age requires bounded interpretation. Group C was substantially younger than Group A, so A–C baseline contrasts remain confounded by limited common age support. The more central A–B comparison had substantial overlap: 22 controls occupied the complete 50–60-year age range of Group A, all 21 contrasts remained after exact-age adjustment, and all remained in the restricted same-age analysis. Age was not systematically associated with outcomes within Group B or with Group C pre–post difference scores after correction. The age-by-group interactions for aggression and responsibility attribution caution against common-slope adjustment alone, but both outcomes remained strongly different in the same-age comparison. Self-selection and other unmeasured characteristics remain unresolved.

Group B served as a valid meditation-naïve comparison cohort for the cross-sectional arm, but it was not reassessed after 4 weeks. The study therefore cannot distinguish the observed Group C pre–post pattern from any variation associated with repeated exposure to the measures, expectancy, the passage of time, or other non-programme influences. Group C was a heterogeneous legally trained cohort that included some serving judicial magistrates, alongside other legal professionals and advanced law trainees. Because the outcomes were standardized hypothetical judgments rather than observed decisions in live cases, the findings may guide future research on judicial education and sentencing practice but do not establish altered real-world judicial decision-making.

### 8.3 Exploratory pathways and retreat subgroup

The indirect-effect reanalysis substantially narrowed the proposed pathways. The anxiety-to-aggression interval was directionally coherent, whereas the aggression-to-RRI pattern was inconsistent mediation or suppression. The SDO-to-RRI interval ended approximately at zero, and the near-perfect positive Free Will–Determinism correlation within Group A indicates severe collinearity. These cross-sectional models identify candidates for temporally ordered testing; they do not establish mechanisms.

The retreat subgroup also warrants a restrained interpretation. Six participants independently undertook a 10-day retreat, but no baseline-adjusted retreat contrast survived FDR correction across the 21 outcomes (minimum *q* = 0.186). This multiplicity-adjusted null result provides no reliable evidence of an incremental retreat association within the pilot. The subgroup was small, self-selected, and non-randomized, so neither benefit nor absence of benefit can be inferred.

### 8.4 Cultural context and normative boundaries

India’s contemplative history may be associated with greater familiarity, credibility, expectancy, and willingness to practice intensively. Cultural receptivity is therefore a plausible moderator rather than a causal explanation. Group A participants reported decades of sustained practice and repeated retreats, while several Group C participants voluntarily increased their practice. Cross-national findings from Norway, Sweden, and the United Kingdom likewise show that national context covaries with free-will beliefs and punishment preferences (Solum et al., 2026). Comparative replication across contemplative and penal cultures is therefore essential.

Religiosity and political orientation remain possible confounders, but their measurement requires cultural care. India’s multiparty environment may not be represented adequately by a single left–right or governing-party–opposition category, and positions on welfare, education, development, punishment, and reform can be cross-cutting. In many Dharmic contexts, religious identity, philosophical worldview, spirituality, and contemplative practice may also overlap. Future Indian studies should use locally validated multidimensional measures rather than imported binary categories.

The normative interpretation is equally limited. Rehabilitation-oriented dispositions can coexist with accountability, proportional sanctions, public-safety conditions, victim-sensitive processes, treatment, supervision, and reintegration; lower punitive severity alone does not demonstrate a superior justice outcome. Individual contemplative education must also remain complementary to structural reform. Treating self-regulation as a substitute for addressing unequal laws, prosecutorial power, deficient mitigation systems, institutional incentives, and carceral harms would reproduce the McMindfulness concern (Purser, 2019).

### 8.5 Limitations

Interpretation should be governed by the limitations summarized in Table 4. Together, they constrain programme-specific attribution, precision, generalizability, and translation from questionnaire and vignette responses to live judicial practice.

**Table 4:** Summary of study limitations and design implications.

Limitation	Interpretive implication	Priority for the next study
Uncontrolled Group C pre–post arm; Group B was not reassessed over 4 weeks	The programme-specific contribution cannot be separated from expectancy, repeated testing, history, regression to the mean, or demand characteristics.	Random assignment with a concurrently reassessed active or wait-list comparison condition.
Self-selected cohorts and incomplete age overlap	Age robustness strengthens the A–B comparison, but personality, religiosity, political orientation, socioeconomic history, and other unmeasured factors remain possible explanations.	Closer age and professional matching, broader recruitment, and culturally validated multidimensional covariates.
*n* = 30 per cohort across 21 outcomes and exploratory pathway models	Effect estimates may be imprecise or inflated; multiplicity correction does not resolve overfitting or correlated outcomes.	Larger preregistered sample with a limited primary-outcome family and adequately powered longitudinal pathway tests.
Self-report, common method, no participant blinding, and no direct expectancy measure	Social desirability, common-method variance, and expectancy may contribute to the observed profile.	Behavioural or externally rated outcomes, expectancy and social-desirability measures, and blinded outcome coding where feasible.
Legally trained but heterogeneous Group C; hypothetical sentencing vignettes	The findings do not directly establish altered sentencing in live cases or generalize to the judiciary as a whole.	Larger judicial samples and behavioural, administrative, or ecologically richer decision outcomes.
Immediate post-assessment and self-reported practice dose	Durability and the relationship between actual dose and outcome patterns remain uncertain.	Objective fidelity measures, prospective dose tracking, and longer-term follow-up.
One Indian region and one contemplative tradition; six outcomes added as an exploratory module	Generalizability is limited, and the supplementary module should remain clearly exploratory.	Multisite cross-cultural replication and prospective specification of all confirmatory outcomes.

### 8.6 Future directions

A definitive next study should be preregistered and adequately powered, with a larger recruitment pool, closer age and professional matching, random assignment, and a concurrently reassessed active or wait-list comparison condition. It should prespecify a limited primary-outcome family, model repeated vignette exposure, measure treatment expectations and social desirability, use objective fidelity indicators and behavioural or externally rated outcomes, and include longer-term follow-up. Multisite recruitment and culturally validated measures of political orientation, religiosity, and related covariates would clarify whether the observed pattern generalizes across settings. The present data are best used to refine that design and to estimate plausible, but potentially inflated, effect ranges.

## 9 Conclusion

This exploratory pilot identified age-robust cross-sectional differences between experienced meditators and meditation-naïve controls and coordinated pre–post differences among legal professionals after a four-week Anapana programme. The direction, magnitude, and coherence of the findings are consistent with a meaningful meditation-related contribution and provide promising hypothesis-generating evidence. Because the intervention was uncontrolled and the cohorts were self-selected, the study cannot estimate the programme-specific causal contribution or establish improved justice outcomes. Replication in larger, preregistered randomized trials with concurrently reassessed active controls, behavioural outcomes, culturally adapted covariates, and follow-up is essential before contemplative training can be recommended for judicial or professional education.

## Data Availability

The datasets generated and analysed during the current study are not publicly available because they contain sensitive participant-level responses (including sentencing judgments by named legal professionals) from a small sample (*N* = 90) recruited in a single city, and are therefore potentially re-identifiable. De-identified data may be made available to qualified researchers from recognised academic institutions on reasonable request to the corresponding author (pragya@allduniv.ac.in), subject to a data-sharing agreement and approval from the Institutional Ethics Committee of the Purshottamdas Savitridevi Cancer Care & Research Centre, Agra. Identifiable participant data will not be shared.
