# Supplementary material for: Meditation, free-will beliefs, and sentencing attitudes: an exploratory pilot study in India
Source: Front Psychol. 2026 Aug 10;17:1865426. doi: 10.3389/fpsyg.2026.1865426 (PMC13500273; doi:10.3389/fpsyg.2026.1865426)

Supplementary Material

Meditation, Free-Will Beliefs, and Sentencing Attitudes: An Exploratory Pilot Study in India

Pragya Mishra, Susan L. Prescott, and Alan C. Logan

Supplementary Methods

S1. Measures and scoring overview

The table below identifies the 21 analysed outcomes. Copyright-restricted questionnaire items are not reproduced. Researchers should consult the cited source instruments and licensing requirements. The four study-authored vignettes and intervention script are reproduced in full below. The dehumanization/MFQ module was initially envisaged for a subsequent study, then incorporated into the ongoing pilot during rolling data collection; Group C completed it before commencing the programme and again at post-assessment.

Outcome	Construct	Items/source	Study score	Source
STAI-6	State anxiety	6	Raw sum, 6–24	Marteau and Bekker (1992)
FWI Free Will	Libertarian/free-will endorsement	5	Subscale total, 5–35	Nadelhoffer et al. (2014)
FWI Determinism	Determinism endorsement	5	Subscale total, 5–35	Nadelhoffer et al. (2014)
FWI Dualism	Mind–body dualism	5	Subscale total, 5–35	Nadelhoffer et al. (2014)
BPAQ-SF	Aggression	12	Published short-form scoring	Bryant and Smith (2001)
SDO-7	Social dominance orientation	16	Published scale scoring	Ho et al. (2015)
BJW	Belief in a just world	7	Published scale scoring	Lipkus (1991)
PJAQ System Confidence	Legal-system confidence	PJAQ subscale	Published scoring	Lecci and Myers (2008)
PJAQ Cynicism	Legal cynicism	PJAQ subscale	Published scoring	Lecci and Myers (2008)
PJAQ Racial Bias	Racialized legal attitudes	PJAQ subscale	Published scoring	Lecci and Myers (2008)
PJAQ Social Justice	Social-justice orientation	PJAQ subscale	Published scoring	Lecci and Myers (2008)
PJAQ Innate Criminality	Essentialist criminality beliefs	PJAQ subscale	Published scoring	Lecci and Myers (2008)
RRI	Rehabilitation/ restorative disposition	4 vignettes	Average disposition score	Study-authored vignettes
Incarceration	Recommended prison duration	4 vignettes	Average years, 0–10	Study-authored vignettes
Responsibility	Responsibility attribution	4 vignettes	Average 1–7 score	Study-authored vignettes
Dehumanization	Blatant dehumanization	1 rating	0–100	Kteily et al. (2015)
MFQ Care	Care foundation	MFQ subscale	0–30	Graham et al. (2011)
MFQ Fairness	Fairness foundation	MFQ subscale	0–30	Graham et al. (2011)
MFQ Loyalty	Loyalty foundation	MFQ subscale	0–30	Graham et al. (2011)
MFQ Authority	Authority foundation	MFQ subscale	0–30	Graham et al. (2011)
MFQ Purity	Purity foundation	MFQ subscale	0–30	Graham et al. (2011)

S2. Participant-facing sentencing instructions

For each scenario, participants were asked to recommend an incarceration term from 0 to 10 years, rate the defendant's responsibility on a 1–7 scale, and select a disposition from custodial to rehabilitative/restorative alternatives. Group C received a legal-register version; Groups A and B received an accessible-language version. The content was designed to be substantively equivalent across registers.

S3. Full vignette text: accessible-language version

1. Aggravated Assault — No Mitigation

Rajesh, 28, got into a fight outside a shop and injured another man who required hospital treatment. He had been drinking; there was no provocation reported. He accepts responsibility. (Legal term: aggravated assault / grievous hurt).

Decide: prison term (0–10 years); responsibility (1–7); disposition (prison / probation with programme / community service with counselling / other).

2. Aggravated Assault — With Mitigation

Rajesh, 28, injured another man in a fight outside a shop; the victim needed hospital care. Rajesh had been drinking. Background information shows that he grew up in a violent household and was assaulted as a teenager. He expresses remorse and wants to break the cycle. (Legal term: aggravated assault / grievous hurt; mitigation presented).

Decide as above.

3. Public Corruption — Serious Bribery

Arun, 36, a junior municipal officer, accepted ₹2,00,000 to approve a commercial building plan that had earlier failed inspection. He admitted guilt after strong evidence emerged and had no prior convictions. (Legal term: criminal misconduct by a public servant / bribery).

Decide: prison term (0–10 years); responsibility (1–7); disposition (prison / financial penalty plus integrity training / probation with compliance monitoring / other).

4. Domestic Violence — Non-fatal Physical Abuse

Suresh, 34, physically assaulted his wife during an argument, causing bruises requiring outpatient care. She does not want to end the marriage but wants him to seek help. (Legal term: assault / domestic-violence context).

Decide: prison term (0–10 years); responsibility (1–7); disposition (short imprisonment / probation plus mandatory behaviour-change programme and protective conditions / other).

S4. Legal-register vignette version for Group C

1. Aggravated Assault — No Mitigation

Rajesh, 28, pleads guilty to aggravated assault (voluntarily causing grievous hurt; BNS offence category) after an altercation outside a shop; the victim required inpatient treatment. No provocation evidence was reported. Alcohol consumption was noted. Submissions included remorse and acceptance of responsibility.

Recommend an imprisonment term and any appropriate custodial, protective, or rehabilitative conditions.

2. Aggravated Assault — With Mitigation

The same principal facts were presented, with mitigation comprising intergenerational exposure to violence, prior victimisation in adolescence, remorse, and willingness to engage in behaviour-change.

Recommend an imprisonment term and any appropriate custodial, protective, or rehabilitative conditions.

3. Public Corruption — Serious Bribery

Arun, 36, a junior municipal officer, is convicted of criminal misconduct by a public servant (bribery) tied to approval of a commercial building plan; consideration ₹2,00,000. Aggravating factor: breach of public trust and concealment until evidence emerged. Mitigating factors: first offence and admission of guilt.

Recommend an imprisonment term and any appropriate custodial, protective, or rehabilitative conditions.

4. Domestic Violence — Non-fatal Physical Abuse

Suresh, 34, is found guilty of assault causing injuries in a domestic setting; the victim required medical care and protective concerns were recorded. The victim seeks behaviour change and safety conditions rather than marital dissolution.

Recommend an imprisonment term and any appropriate custodial, protective, or rehabilitative conditions.

S5. Core Anapana instructional script

The initial ethics protocol contained the following simplified secular script, which formed part of the instructional framework. The final four-week programme also used written Goenka-tradition Anapana instructions and 20–25-minute audio guidance, followed by approximately 10-minute reinforcement recordings.

“Please assume a comfortable seated position. You may close your eyes if this feels appropriate, or maintain a soft downward gaze. Direct your attention to the natural rhythm of your breathing. Observe the sensation of breath entering and leaving your body without attempting to modify its pattern. When you notice your attention has shifted to thoughts, plans, or concerns—a natural occurrence—simply acknowledge this movement of mind and gently redirect awareness to the breath without self-

criticism. Continue resting awareness on the body breathing, sitting, and being present. When you feel ready, deepen the breath slightly and, if your eyes were closed, allow them to open gently.”

Supplementary Statistical Tables

Supplementary Table S1. Complete Descriptive Statistics and Omnibus Group Comparisons

Outcome	A M (SD)	B M (SD)	C-pre M (SD)	F(2,87)	p	FDR q	η²	d A–B	d A–C	d B–C	C-post M (SD)
STAI-6 (anxiety)	8.40 (1.52)	16.80 (2.20)	15.80 (1.61)	194.30	< .001	< .001	0.817	-4.44	-4.73	+0.52	12.60 (1.10)
FWI free will	22.30 (4.11)	28.70 (2.56)	27.50 (1.94)	38.23	< .001	< .001	0.468	-1.87	-1.62	+0.53	25.30 (0.95)
FWI determinism	24.50 (3.25)	15.50 (1.83)	16.80 (1.27)	137.31	< .001	< .001	0.759	+3.41	+3.12	-0.82	18.70 (1.44)
FWI dualism	26.50 (2.36)	23.30 (2.32)	22.30 (1.73)	31.09	< .001	< .001	0.417	+1.37	+2.03	+0.49	23.50 (2.11)
BPAQ-SF (aggression)	23.30 (5.10)	35.70 (2.71)	31.20 (2.31)	91.71	< .001	< .001	0.678	-3.04	-2.00	+1.79	27.10 (1.77)
SDO-7	37.60 (7.27)	62.50 (4.00)	59.50 (3.61)	203.29	< .001	< .001	0.824	-4.25	-3.82	+0.79	54.20 (2.71)
BJW	22.50 (2.60)	33.00 (2.59)	29.30 (2.51)	129.47	< .001	< .001	0.749	-4.05	-2.66	+1.45	27.90 (1.88)
PJAQ system confidence	15.30 (1.70)	24.00 (1.93)	26.50 (2.03)	289.32	< .001	< .001	0.869	-4.78	-5.97	-1.26	24.60 (1.28)
PJAQ cynicism	15.20 (1.63)	22.70 (2.07)	20.30 (2.09)	116.95	< .001	< .001	0.729	-4.03	-2.73	+1.15	18.40 (1.33)
PJAQ racial bias	8.60 (1.79)	15.30 (1.86)	14.60 (1.57)	133.74	< .001	< .001	0.755	-3.67	-3.56	+0.41	12.80 (1.32)
PJAQ social justice	15.70 (1.68)	10.80 (1.61)	12.50 (1.43)	74.59	< .001	< .001	0.632	+2.98	+2.05	-1.12	13.70 (1.66)
PJAQ innate criminality	7.60 (1.69)	17.50 (1.76)	14.80 (1.94)	242.83	< .001	< .001	0.848	-5.74	-3.96	+1.46	12.40 (1.33)
RRI	4.40 (0.61)	2.80 (0.39)	3.20 (0.29)	100.98	< .001	< .001	0.699	+3.11	+2.49	-1.15	4.10 (0.58)
Incarceration	1.70 (0.77)	4.70 (0.65)	3.60 (0.57)	153.91	< .001	< .001	0.780	-4.20	-2.80	+1.79	2.80 (0.39)
Responsibility	4.20 (0.58)	5.60 (0.33)	5.40 (0.35)	89.82	< .001	< .001	0.674	-2.95	-2.49	+0.58	4.90 (0.21)
Dehumanization	13.97 (5.19)	33.77 (6.32)	32.43 (7.99)	84.39	< .001	< .001	0.660	-3.43	-2.74	+0.19	20.57 (8.48)
MFQ care	25.20 (2.28)	19.63 (2.47)	19.23 (2.10)	63.80	< .001	< .001	0.595	+2.34	+2.72	+0.17	22.63 (2.58)
MFQ fairness	24.63 (2.30)	19.53 (2.36)	19.90 (2.06)	48.31	< .001	< .001	0.526	+2.19	+2.17	-0.17	22.57 (1.83)
MFQ loyalty	12.00 (1.89)	16.97 (2.61)	17.23 (2.39)	48.62	< .001	< .001	0.528	-2.18	-2.43	-0.11	13.73 (2.73)
MFQ authority	12.83 (1.91)	17.20 (2.02)	16.93 (2.53)	38.06	< .001	< .001	0.467	-2.22	-1.83	+0.12	13.77 (2.69)
MFQ purity	11.50 (1.81)	16.77 (2.69)	16.57 (2.84)	43.20	< .001	< .001	0.498	-2.30	-2.13	+0.07	12.87 (3.14)

All omnibus raw p values and FDR q values were below .001 and below the Bonferroni threshold .00238. C-post descriptives are included for completeness, although the omnibus test compares A, B, and C-pre.

Supplementary Table S2. Age-Adjusted and Overlapping-Age Group A–B Analyses. HC3-robust Wald tests were used. All adjusted group contrasts and all same-age contrasts were below p = .00238. Exact p and q values are retained in the analysis files. Age-by-group interactions survived FDR correction for aggression and responsibility attribution only.

Outcome	Adjusted standardized contrast	HC3 95% CI	p	FDR q	Same-age d (50–60 years)	Age×group p	FDR q
STAI-6 (anxiety)	1.771	[1.530, 2.011]	< .001	< .001	+4.19	.272	.715
FWI free will	1.329	[0.955, 1.703]	< .001	< .001	+1.77	.487	.902

Outcome	Adjusted standardized contrast	HC3 95% CI	p	FDR q	Same-age d (50–60 years)	Age×group p	FDR q
FWI determinism	1.750	[1.489, 2.011]	< .001	< .001	+3.31	.785	.992
FWI dualism	1.170	[0.725, 1.616]	< .001	< .001	+1.37	.889	.992
BPAQ-SF (aggression)	1.565	[1.289, 1.841]	< .001	< .001	+2.76	< .001	.015
SDO-7	1.762	[1.536, 1.988]	< .001	< .001	+3.91	.849	.992
BJW	1.741	[1.502, 1.979]	< .001	< .001	+3.82	.218	.654
PJAQ system confidence	1.848	[1.631, 2.065]	< .001	< .001	+4.75	.119	.462
PJAQ cynicism	1.747	[1.488, 2.006]	< .001	< .001	+3.76	.132	.462
PJAQ racial bias	1.691	[1.425, 1.957]	< .001	< .001	+3.42	.644	.902
PJAQ social justice	1.680	[1.369, 1.992]	< .001	< .001	+2.92	.973	.992
PJAQ innate criminality	1.827	[1.647, 2.007]	< .001	< .001	+5.60	.951	.992
RRI	1.755	[1.463, 2.046]	< .001	< .001	+3.08	.014	.096
Incarceration	1.777	[1.547, 2.007]	< .001	< .001	+4.04	.528	.902
Responsibility	1.622	[1.325, 1.918]	< .001	< .001	+2.83	.004	.047
Dehumanization	1.734	[1.468, 2.000]	< .001	< .001	+3.60	.565	.902
MFQ care	1.486	[1.128, 1.843]	< .001	< .001	+2.37	.351	.820
MFQ fairness	1.505	[1.151, 1.860]	< .001	< .001	+2.21	.056	.295
MFQ loyalty	1.562	[1.211, 1.912]	< .001	< .001	+2.62	.613	.902
MFQ authority	1.536	[1.181, 1.891]	< .001	< .001	+2.41	.608	.902
MFQ purity	1.543	[1.192, 1.894]	< .001	< .001	+2.35	.992	.992

Supplementary Table S3. Exploratory Single-Mediator Decomposition (20,000 fixed-seed, bias-corrected bootstrap resamples; seed = 1865426)

Model	a	b	Total c	Direct c′	Indirect ab	Bias-corrected 95% CI	Interpretation
SDO-7 → RRI	-0.907	+0.275	+0.845	+1.094	-0.249	[-0.551, +0.004]	Boundary interval; not reliably distinguishable from zero
SDO-7 → incarceration	-0.907	+0.034	-0.906	-0.875	-0.031	[-0.309, +0.201]	CI includes zero
BPAQ-SF → RRI	-0.839	+0.439	+0.845	+1.213	-0.368	[-0.610, -0.133]	Inconsistent indirect effect / suppression
BPAQ-SF → incarceration	-0.839	+0.119	-0.906	-0.806	-0.100	[-0.327, +0.123]	CI includes zero
BJW → responsibility	-0.900	+0.198	-0.832	-0.654	-0.178	[-0.489, +0.122]	CI includes zero
FWI determinism → responsibility	+0.867	+0.182	-0.832	-0.990	+0.158	[-0.144, +0.462]	CI includes zero
PJAQ innate criminality → incarceration	-0.946	+0.209	-0.906	-0.708	-0.197	[-0.522, +0.113]	CI includes zero

Model	a	b	Total c	Direct c′	Indirect ab	Bias-corrected 95% CI	Interpretation
STAI-6 → BPAQ-SF	-0.914	+0.406	-0.839	-0.469	-0.371	[-0.645, -0.049]	Directionally consistent indirect effect
SDO-7 → responsibility	-0.907	+0.151	-0.832	-0.695	-0.137	[-0.431, +0.133]	CI includes zero
PJAQ racial bias → incarceration	-0.881	+0.229	-0.906	-0.704	-0.202	[-0.443, +0.026]	CI includes zero
Dehumanization → RRI	-0.867	+0.045	+0.845	+0.884	-0.039	[-0.285, +0.146]	CI includes zero
MFQ care → incarceration	+0.766	+0.100	-0.906	-0.983	+0.077	[-0.031, +0.186]	CI includes zero
MFQ fairness → RRI	+0.744	+0.077	+0.845	+0.787	+0.057	[-0.106, +0.219]	CI includes zero
Dehumanization → incarceration	-0.867	-0.057	-0.906	-0.955	+0.050	[-0.134, +0.217]	CI includes zero
Dehumanization → responsibility	-0.867	-0.046	-0.832	-0.872	+0.040	[-0.180, +0.244]	CI includes zero
MFQ care → RRI	+0.766	-0.077	+0.845	+0.904	-0.059	[-0.265, +0.092]	CI includes zero
MFQ loyalty → incarceration	-0.743	+0.023	-0.906	-0.888	-0.017	[-0.137, +0.080]	CI includes zero
MFQ loyalty → responsibility	-0.743	+0.022	-0.832	-0.815	-0.017	[-0.188, +0.130]	CI includes zero

Group coded A = 1 and B = 0. Models are cross-sectional and do not establish temporal or causal mediation. The SDO-7 → RRI interval ended approximately at zero and is treated as a boundary result rather than a robust indirect effect.

Supplementary Table S4. Baseline-Adjusted Retreat Contrasts

Outcome	Adjusted retreat coefficient	HC3 95% CI	Nominal p	FDR q
STAI-6 (anxiety)	+0.131	[-0.650, +0.912]	0.742	0.944
FWI free will	+0.044	[-0.617, +0.706]	0.896	0.946
FWI determinism	+0.117	[-0.576, +0.810]	0.741	0.944
FWI dualism	+0.144	[-0.250, +0.537]	0.474	0.766
BPAQ-SF (aggression)	+0.483	[+0.033, +0.933]	0.0354	0.186
SDO-7	-0.347	[-0.825, +0.131]	0.155	0.541
BJW	-0.146	[-0.517, +0.224]	0.439	0.766
PJAQ system confidence	+0.020	[-0.446, +0.487]	0.932	0.946
PJAQ cynicism	+0.053	[-0.456, +0.563]	0.838	0.946
PJAQ racial bias	-0.471	[-0.893, -0.049]	0.0288	0.186
PJAQ social justice	-0.315	[-0.573, -0.058]	0.0165	0.186
PJAQ innate criminality	+0.083	[-0.436, +0.602]	0.754	0.944
RRI	-0.010	[-0.305, +0.285]	0.946	0.946
Incarceration	+0.141	[-0.196, +0.478]	0.411	0.766
Responsibility	-0.346	[-0.725, +0.033]	0.0734	0.308
Dehumanization	-0.449	[-1.180, +0.281]	0.228	0.683
MFQ care	+0.453	[-0.456, +1.362]	0.329	0.717
MFQ fairness	+0.972	[+0.069, +1.876]	0.0349	0.186
MFQ loyalty	-0.414	[-1.238, +0.411]	0.325	0.717
MFQ authority	-0.221	[-1.670, +1.227]	0.765	0.944
MFQ purity	-0.493	[-1.508, +0.522]	0.341	0.717

Retreat n = 6; non-retreat n = 24. No outcome survived FDR correction.

Supplementary Table S5A. Age–Outcome Associations Within Group B (n = 30)

Outcome	Pearson r	p	FDR q	Spearman ρ	p	FDR q
STAI-6 (anxiety)	-0.120	.529	.820	-0.016	.934	.980
FWI free will	-0.076	.690	.852	-0.062	.746	.898
FWI determinism	-0.158	.405	.820	-0.143	.452	.862
FWI dualism	-0.103	.586	.820	-0.091	.631	.898
BPAQ-SF (aggression)	-0.251	.180	.648	-0.204	.279	.681
SDO-7	-0.247	.189	.648	-0.288	.123	.681
BJW	-0.281	.133	.648	-0.281	.132	.681
PJAQ system confidence	+0.166	.380	.820	+0.264	.158	.681
PJAQ cynicism	-0.233	.216	.648	-0.250	.183	.681
PJAQ racial bias	-0.268	.153	.648	-0.204	.279	.681
PJAQ social justice	-0.093	.625	.820	-0.176	.352	.739
PJAQ innate criminality	-0.304	.102	.648	-0.202	.283	.681
RRI	-0.188	.321	.820	-0.088	.642	.898
Incarceration	-0.055	.773	.898	+0.087	.649	.898
Responsibility	+0.111	.561	.820	+0.199	.292	.681
Dehumanization	+0.017	.931	.931	+0.056	.770	.898
MFQ care	+0.041	.829	.898	+0.005	.981	.981
MFQ fairness	+0.035	.856	.898	+0.077	.685	.898
MFQ loyalty	+0.261	.164	.648	+0.227	.228	.681
MFQ authority	+0.122	.521	.820	+0.066	.731	.898
MFQ purity	+0.097	.610	.820	+0.041	.830	.917

Note. FDR correction was applied across all 21 outcomes within each correlation family. No association survived correction.

Supplementary Table S5B. Age–Change Associations Within Group C (n = 30)

Outcome	Pearson r	p	FDR q	Spearman ρ	p	FDR q
STAI-6 (anxiety)	-0.198	.293	.441	-0.091	.631	.779
FWI free will	+0.200	.290	.441	+0.132	.487	.763
FWI determinism	-0.219	.245	.441	-0.184	.330	.763
FWI dualism	-0.367	.046	.441	-0.139	.464	.763
BPAQ-SF (aggression)	-0.324	.081	.441	-0.189	.316	.763
SDO-7	+0.299	.109	.441	+0.099	.603	.779
BJW	+0.052	.786	.956	+0.132	.487	.763
PJAQ system confidence	+0.221	.240	.441	+0.251	.181	.763
PJAQ cynicism	+0.249	.185	.441	+0.276	.140	.763
PJAQ racial bias	+0.000	1.000	1.000	+0.165	.385	.763
PJAQ social justice	+0.356	.053	.441	+0.436	.016	.337
PJAQ innate criminality	-0.207	.271	.441	+0.081	.672	.783
RRI	-0.026	.892	.956	-0.115	.545	.763
Incarceration	-0.262	.162	.441	+0.019	.919	.919
Responsibility	-0.209	.269	.441	-0.174	.358	.763
Dehumanization	-0.033	.861	.956	+0.122	.521	.763
MFQ care	-0.198	.294	.441	-0.168	.375	.763
MFQ fairness	-0.176	.353	.494	-0.190	.314	.763
MFQ loyalty	-0.087	.649	.852	+0.029	.878	.919
MFQ authority	-0.318	.086	.441	-0.215	.253	.763
MFQ purity	+0.021	.911	.956	-0.049	.798	.882

Note. FDR correction was applied across all 21 outcomes within each correlation family. No association survived correction.

Supplementary Figures

Supplementary Figure S1. Violin, box, and jitter plots for the 15 core outcomes across Groups A, B, C-pre, and C-post. These plots permit inspection of distribution shape, dispersion, and potential floor or ceiling patterns.

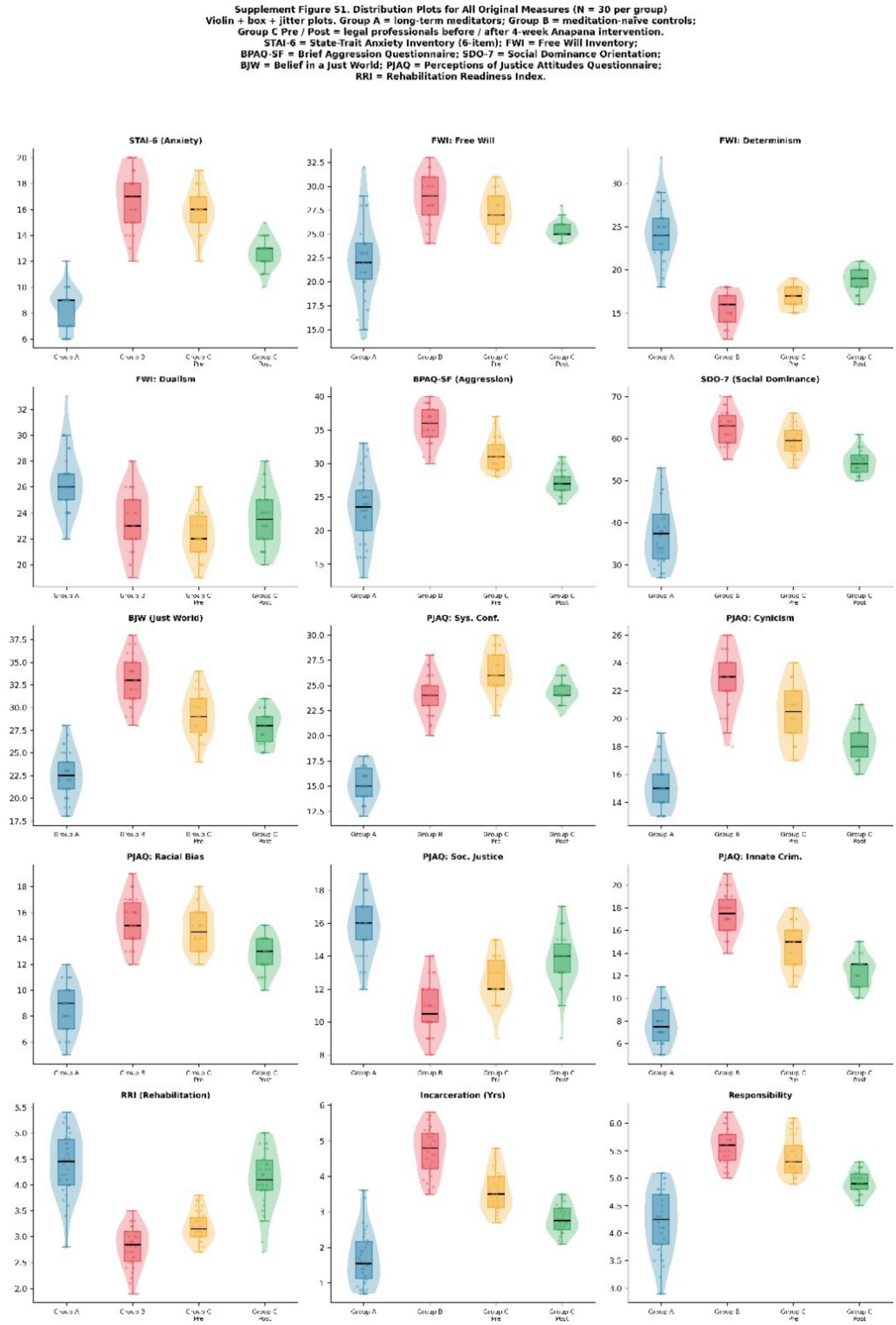

Supplementary Figure S2. Violin, box, and jitter plots for dehumanization and the five MFQ subscales.

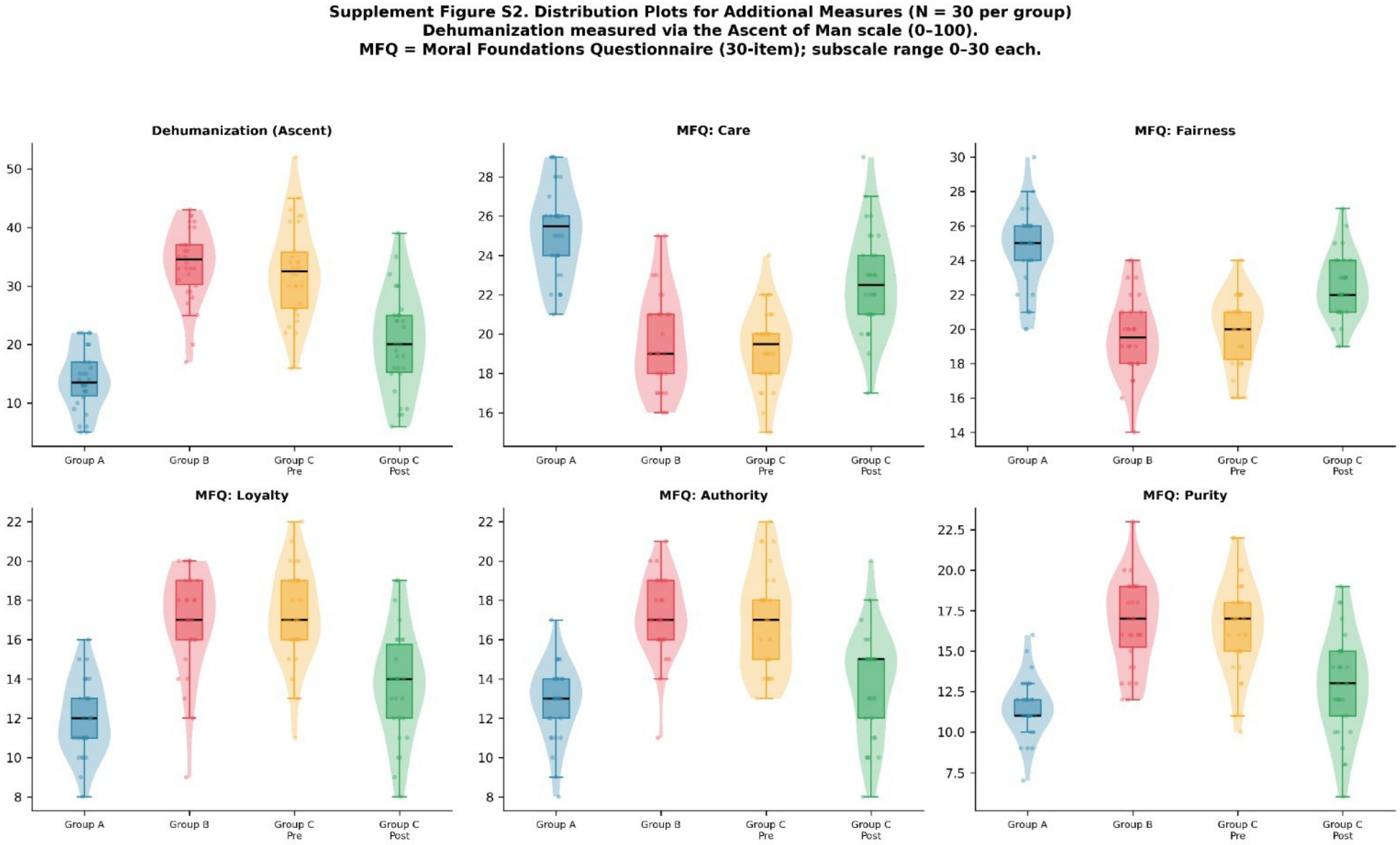

Supplementary Figure S3A. Participant-level pre–post slopes for the 15 core outcomes, displayed by retreat status. Retreat participation was self-selected.

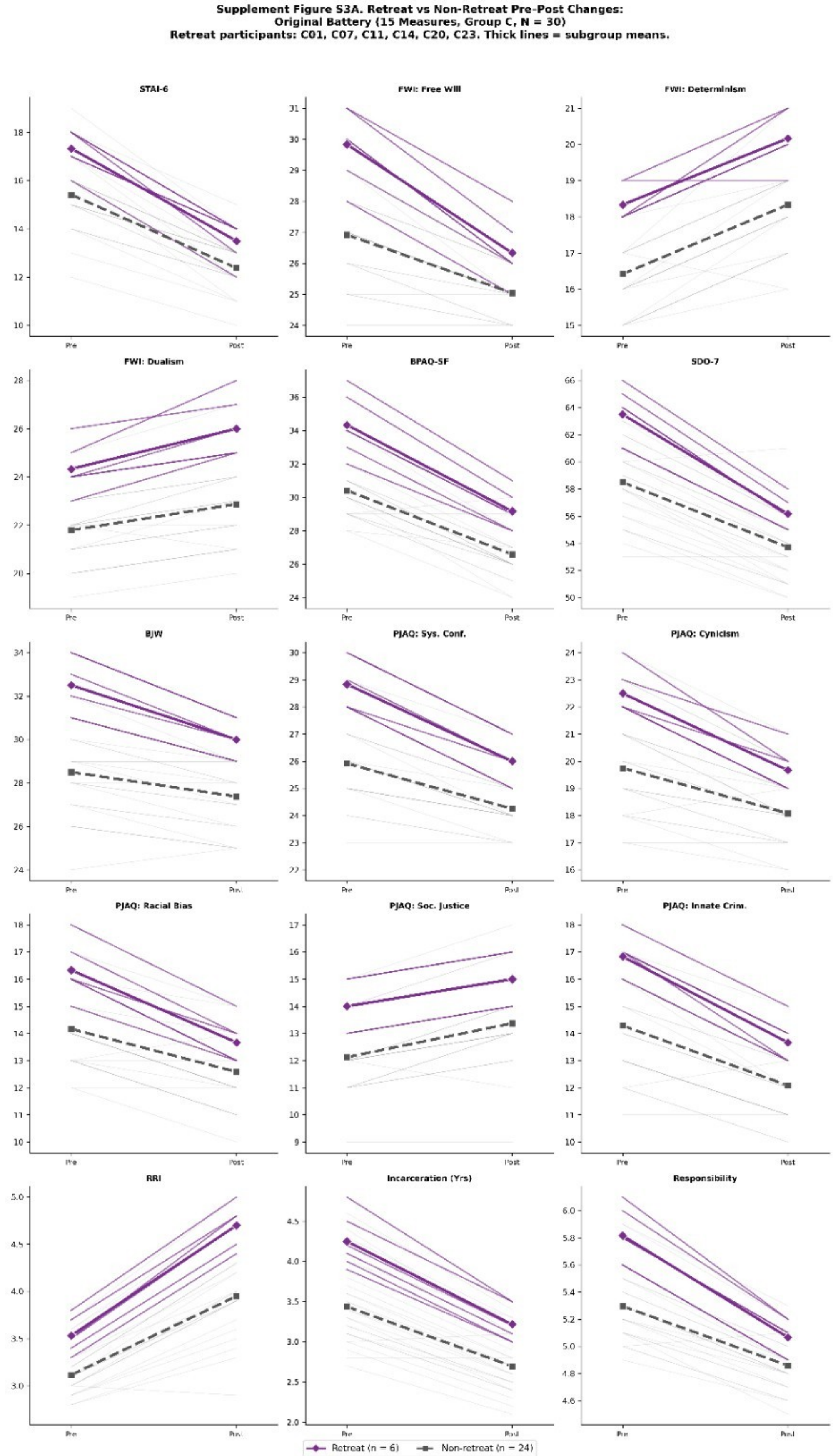

Supplementary Figure S3B. Participant-level pre–post slopes for the six exploratory supplementary outcomes, displayed by retreat status.

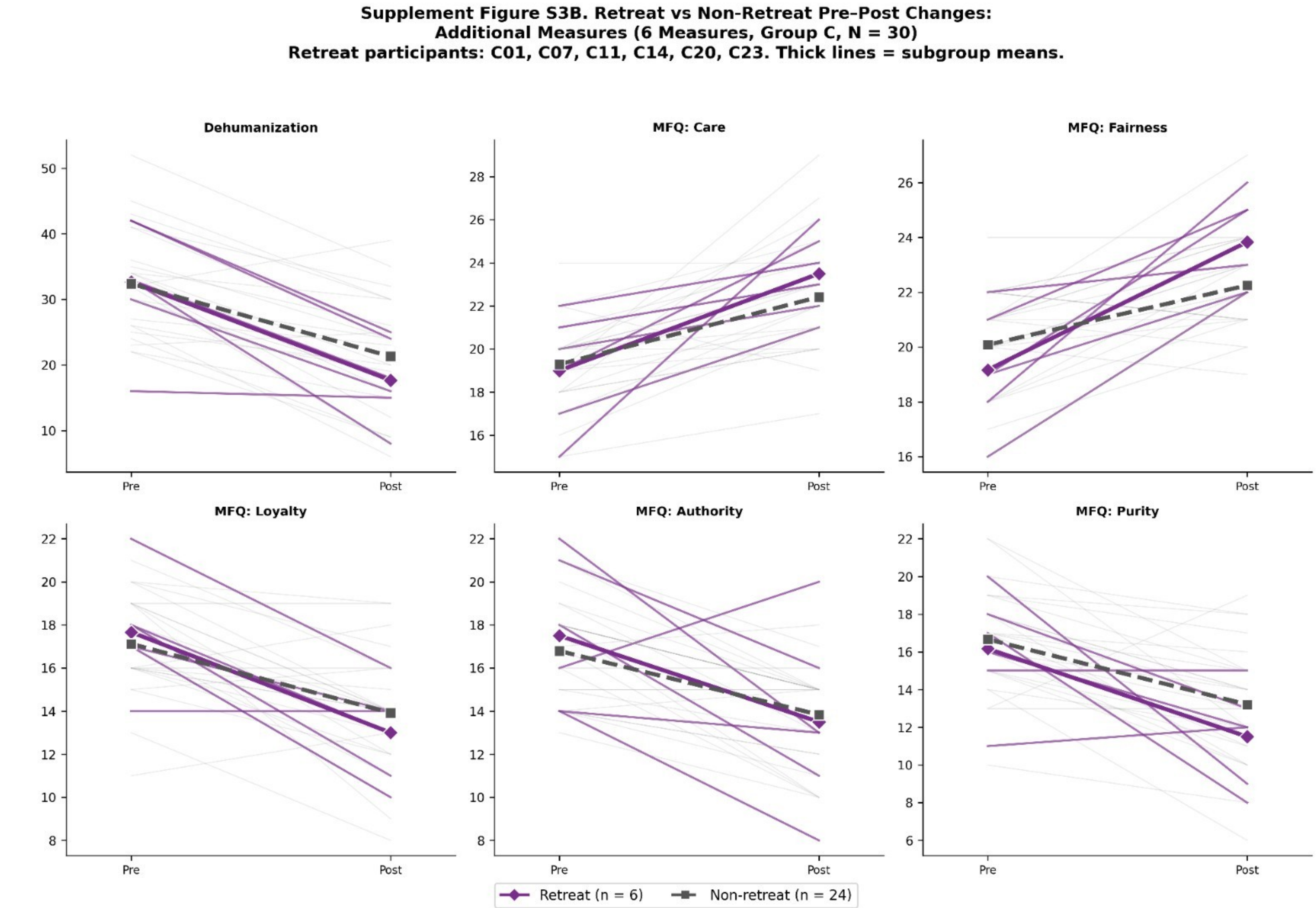

Supplementary Figure S4. Pearson correlations among directionally aligned change scores. Positive values indicate that two outcomes tended to move together toward the observed Group A profile. Correlation does not establish temporal ordering or mediation.

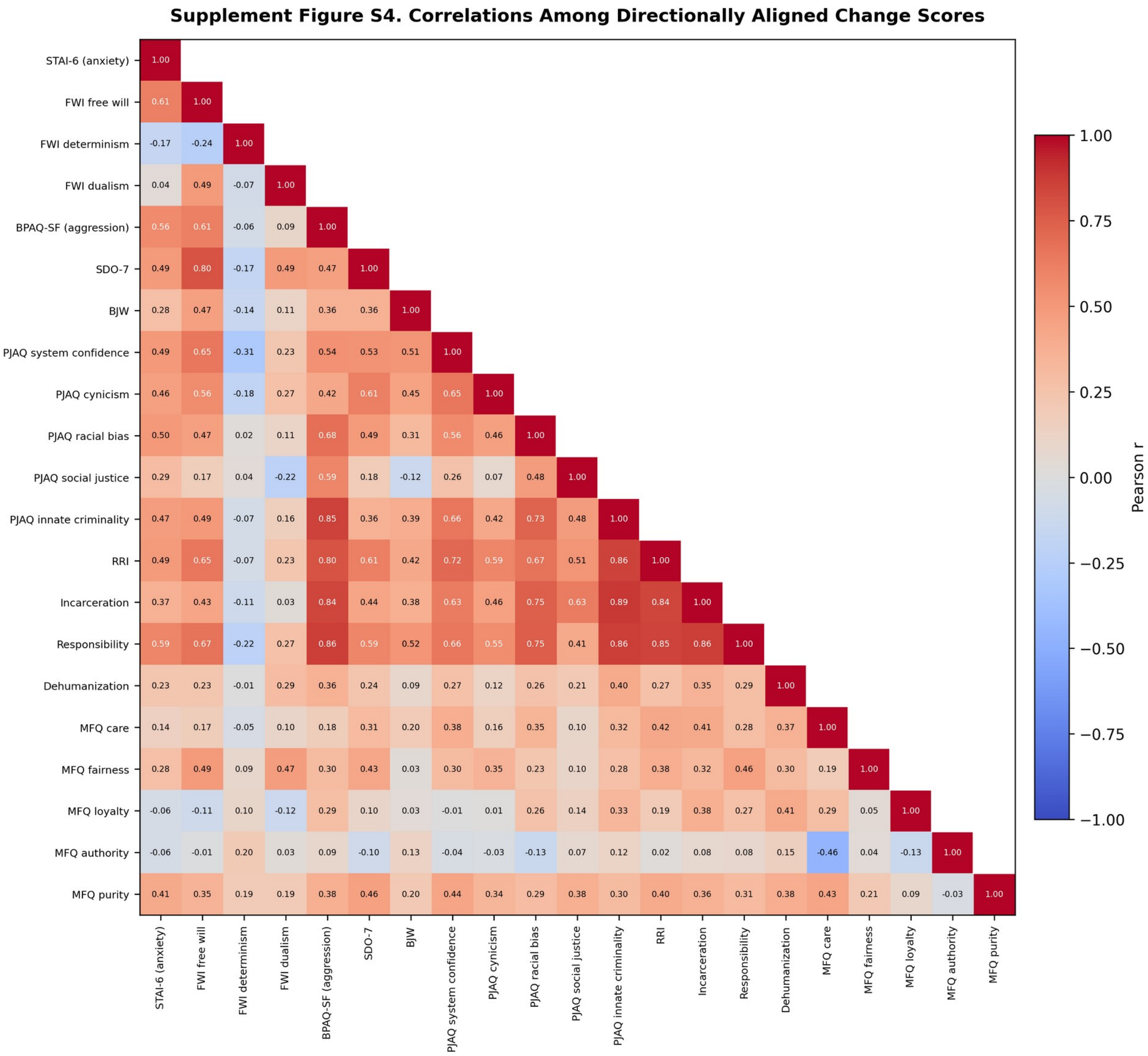

Supplementary Figure S5. Percentage of the 21 outcomes moving in the hypothesised direction for each Group C participant. Hatched bars identify retreat participants; the black outline identifies C21. The index describes direction only and is not a clinical responder classification.

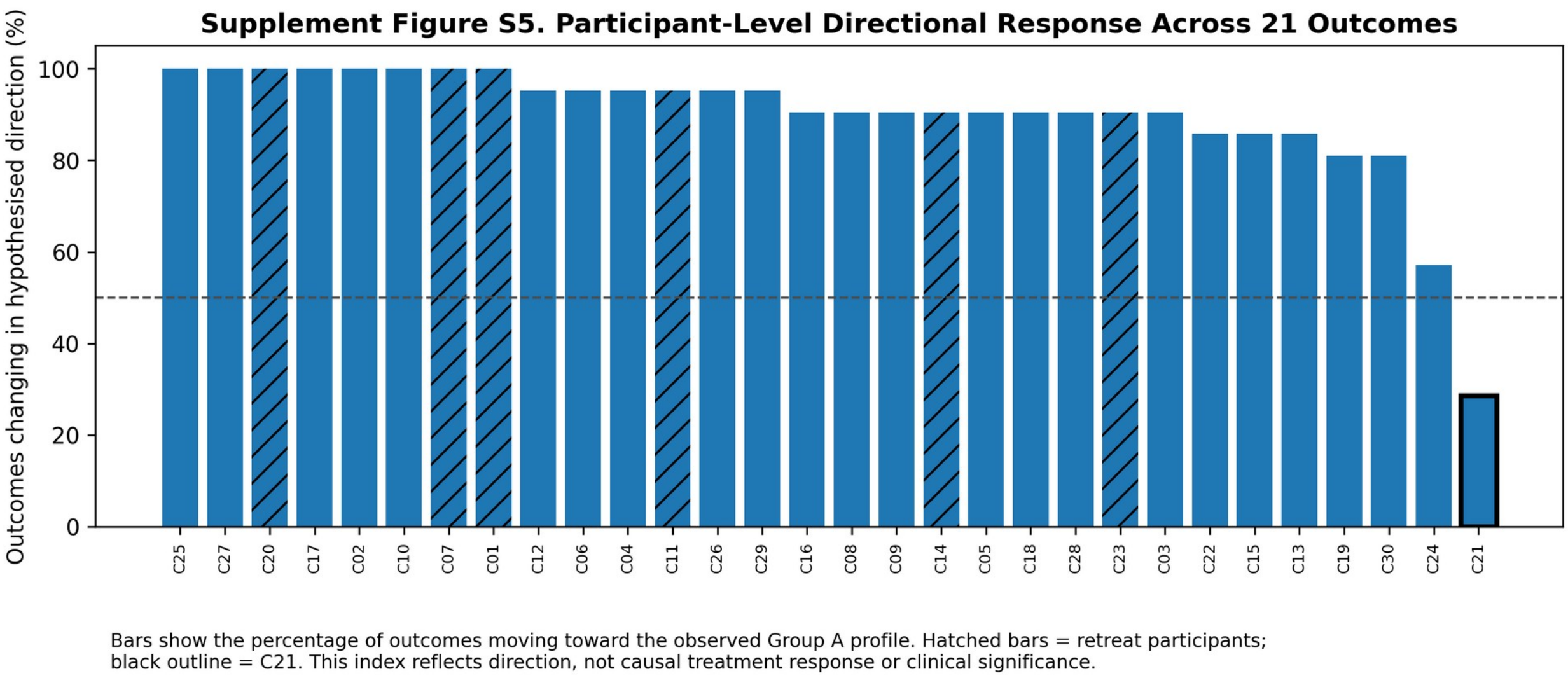

Supplementary Figure S6. Descriptive heatmap of directionally aligned group means across 21 outcomes. The heatmap is a profile visualization, not an inferential test; “higher” indicates closer alignment with the observed Group A direction rather than a universal measure of compassion.

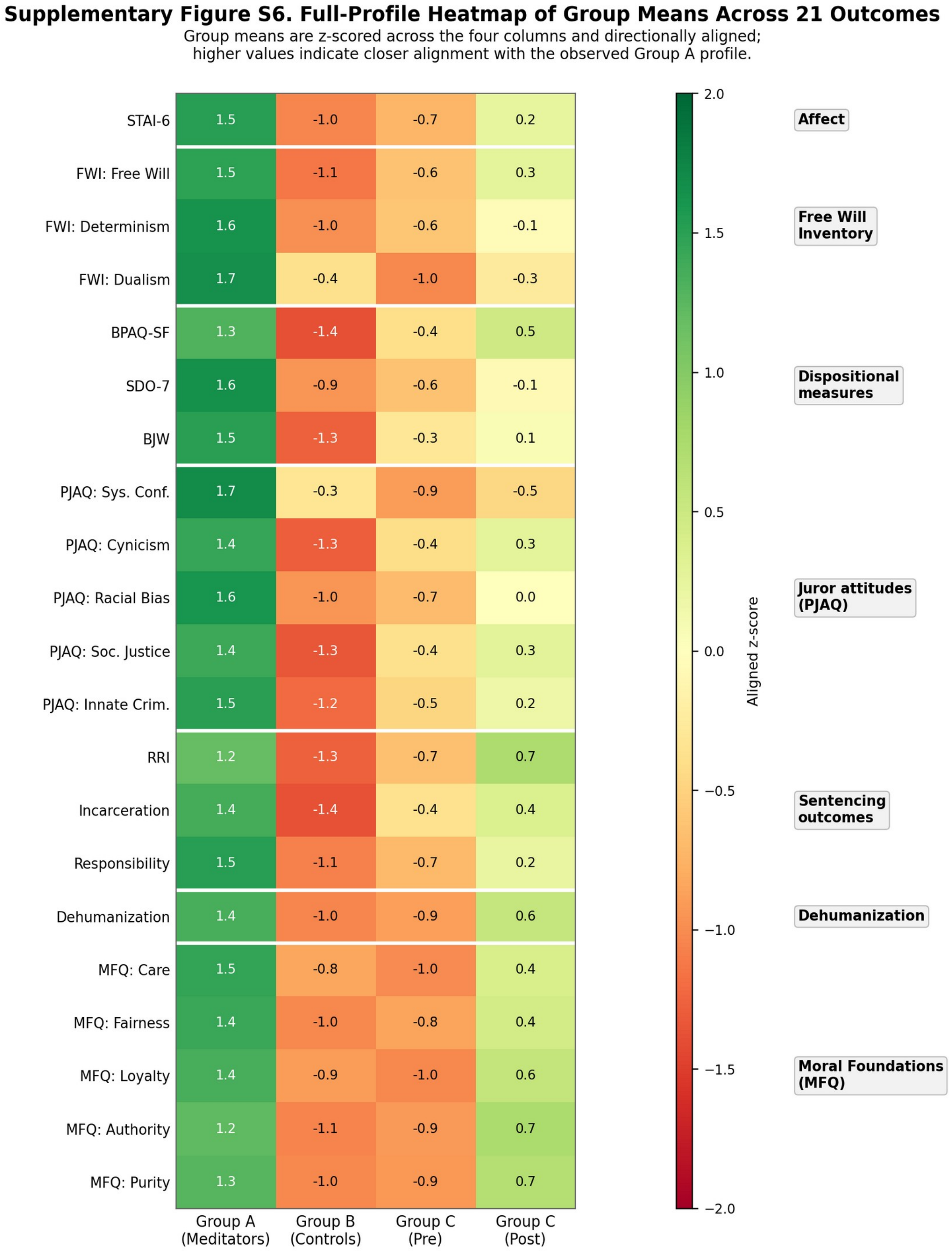

Supplement: Supplementary file 1 [file Data_Sheet_1.PDF]
